# UAV-Induced Measurement Bias in Air Pollution Sensing: A Physics-Informed Framework for Characterization, Sensor Placement, and Correction

**DOI:** 10.3390/s26196178

**Published:** 2026-09-29

**Authors:** Farhad Samadzadegan, Erfan Ghorbani

**Affiliations:** Department of Photogrammetry and Remote Sensing, School of Surveying and Geospatial Engineering, College of Engineering, University of Tehran, Tehran P.O. Box 11365-4563, Iran; samadz@ut.ac.ir

**Keywords:** UAV-based air-quality monitoring, rotor-induced turbulence, measurement bias, sensor placement strategy

## Abstract

**Highlights:**

**What are the main findings?**

- UAV-based air pollution measurements are systematically influenced by rotor-induced aerodynamic disturbances and UAV motion.
- Measurement effects vary with sensor location, flight condition, and pollutant characteristics.

**What are the implications of the main findings?**

- UAV platform-induced effects should be considered when designing and interpreting air pollution monitoring campaigns.
- The findings support more reliable sensor placement and measurement strategies for multi-rotor UAVs.

**Abstract:**

Unmanned aerial vehicles (UAVs) offer flexible, three-dimensional access for air pollution monitoring, but rotor-induced aerodynamic disturbance can bias onboard sensor readings, an effect not yet well characterized for multi-rotor platforms. This paper presents a physics-informed framework unifying rotor wake characterization, turbulence zone classification, pollutant sensitivity analysis, sensor placement strategy, and bias correction into one design tool. Grounded in actuator disk momentum theory and literature-constrained relationships, it predicts or bounds measurement bias across nine pollutants and multiple rotor configurations, quantitative for seven sensor classes and provisional for O_3_ and VOCs. It also introduces a four-zone contamination classification and a two-stage bias correction for motion-induced and environmental factors. Hover and constant-speed co-location flight tests on a 21-inch quadrotor, comparing a UAV-mounted sensor against a fixed reference under Zone 4 conditions, support these predictions: observed bias (3–10% in hover; 6–15% at 5 m/s, across NO_2_, CO, O_3_, PM_10_, and PM_2.5_) was directionally consistent with the predictions, though correlation dropped as low as r = 0.44 in forward flight, below typical validation thresholds. The tests thus support the framework’s qualitative trends more than quantitative agreement, with wind-tunnel and multi-speed validation as natural next steps. The framework offers a reproducible foundation for UAV sensor integration design, uncertainty estimation, and campaign planning.

## 1. Introduction

Air pollution remains one of the most pressing global public health challenges. The World Health Organization estimates that ambient air pollution contributes to about 4.2 million premature deaths annually [1]. More recent global burden-of-disease estimates put ambient and household air pollution combined at about 8.1 million deaths in 2021, reinforcing the need for spatially resolved, three-dimensional air quality observation [2]. Fine particulate matter, ozone, and nitrogen dioxide are of particular concern in urban and industrial settings, where concentrations vary sharply with height, distance from source, and local meteorology. Conventional ground-based monitoring networks give temporally continuous measurements but are limited in spatial resolution: they cannot characterize the three-dimensional distribution of pollutants in urban canopy layers, industrial emission plumes, or complex terrain [3]. Satellite remote sensing offers synoptic coverage but is constrained by coarse spatial resolution, atmospheric column averaging, and cloud contamination [4].

Unmanned aerial vehicles (UAVs) have emerged as effective complementary platforms for atmospheric monitoring, offering relatively low operational cost, programmable sampling trajectories, and the capability for three-dimensional spatial coverage [5,6,7,8]. These characteristics make UAVs well suited to applications that conventional platforms handle poorly, such as mapping vertical pollutant gradients above urban street canyons (subject to the rotor-induced mixing caveat detailed in Section 5.6.1, which bounds this application under low-wind, stable-atmosphere conditions), characterizing point-source industrial plumes at close range, and accessing terrain or airspace where fixed monitoring infrastructure is impractical. Realizing this potential depends on the accuracy of the onboard sensors themselves, which brings the platform’s own aerodynamic behavior into focus.

Multi-rotor platforms offer hovering and low-altitude sampling that fixed-wing designs lack, making them attractive for spatially targeted air quality monitoring at a fixed location or along a slow vertical profile. But this comes with a fundamental aerodynamic limitation: the same rotors that enable hovering also generate a flow field that can disturb the air being sampled. An onboard sensor may then record a mixture of ambient air and rotor-induced downwash rather than the true local concentration [9,10,11,12]. Prior work has examined rotor aerodynamics, UAV-based pollutant sampling, and low-cost sensor calibration largely in isolation, leaving open how these effects combine to bias measurements and how that bias can be anticipated and corrected. This paper’s physics-informed framework addresses that question.

This paper traces the full chain from rotor aerodynamics to sensor-level measurement bias. Starting from actuator disk momentum theory, the framework characterizes the turbulence sources generated by multi-rotor platforms, classifies the resulting sensor contamination zones, and quantifies pollutant-specific sensitivity across nine target species. These predictions inform practical sensor placement guidelines and a two-stage bias correction formulation, which are then evaluated against hover and constant-speed flight tests on a reference quadrotor platform.

## 2. Related Work

The literature on UAV-based air quality monitoring is organized here into four categories: platform-level deployment studies that establish feasibility and application context; aerodynamic characterizations of propeller downwash and rotor wake structure; investigations of how induced airflow affects sensor accuracy; and studies addressing sensor placement strategies. This ordering follows the analytical chain examined later in this paper. Reviewing the literature in this sequence shows that each component has largely been studied in isolation.

UAV deployment for atmospheric monitoring has expanded substantially. Villa et al. [6] reviewed 60 early studies, establishing the performance envelope of multi-rotor platforms; a PRISMA meta-analysis of 241 papers [5] documented advances in sensor miniaturization, platform diversity, and AI/IoT data processing; neither gave a quantitative methodology for estimating or correcting rotor-induced bias. Ref. [7] illustrated the practical stakes: UAV-measured PM_2.5_ at 100 m altitude in Delhi substantially exceeded ground-level readings, and uncorrected rotor bias in such deployments could mask genuine atmospheric gradients. A review of 94 studies across diverse emission sources [8] similarly flagged propeller-induced downwash and sensor calibration as unresolved, calling for standardized protocols—corroborating the gap targeted here.

### 2.1. Propeller Downwash and Rotor Wake Aerodynamics

Propeller-induced downwash has been characterized extensively in the agricultural drone and helicopter literature, yet these findings remain disconnected from sensor bias estimation. Ref. [9] quantified the downwash flow structure of a single rotor blade via constant-temperature anemometry, showing turbulence intensity depends on blade tip velocity and radial position; ref. [10] extended this across the full RPM range using combined thrust measurement and CFD; and ref. [11] showed via wind tunnel and CFD that close rotor separation produces complex interference affecting thrust and wake structure. A combined theoretical/CFD/hot-wire study derived a closed-form solution for propeller-induced hover velocity to guide aerosol sensor placement on three platforms [12], confirming the problem’s practical importance but restricted to hover conditions on individual platforms. These characterizations still stop short of linking wake structure quantitatively to pollutant-specific bias.

### 2.2. Effects of Turbulence on Sensor Accuracy

Several studies quantify sensor-class sensitivity to turbulence, though the findings remain fragmented. Ref. [13] found turbulence intensity above 10% increases optical particle-counting errors by 8–15%; ref. [14] found wind-driven flow across electrochemical NO_2_ membranes introduces 5–25% bias at 2–10 m/s; ref. [15] showed deviations from isokinetic sampling cause 10–40% collection-efficiency errors for PM; and ref. [16] identified downwash, mounting location, and isokinetic sampling as the three critical determinants of measurement quality, without analytical error estimates. Together these establish the physical basis for pollutant-class sensitivity but stop short of a cross-pollutant bias framework. A decade-spanning review of ML-based sensor calibration further shows research has focused on environmental confounders (humidity, temperature, cross-sensitivity), leaving platform-induced aerodynamic disturbance underexplored [17].

### 2.3. Sensor Placement Strategies

Few experimental studies examine sensor placement on UAVs; those that do confirm the importance of mounting geometry but remain platform-specific. Ref. [18] quantified propeller-induced concentration errors against a tower reference and proposed an empirical correction; ref. [19] showed sensor position above the rotor plane was the primary determinant of PM and O_3_ measurement quality; and ref. [20] achieved 5–12% agreement for CH_4_ with an optimized inlet under stable conditions. These studies remain platform-specific and stop short of a unified methodology spanning propeller sizes, rotor configurations, and pollutant classes. A comparative summary of these studies is provided in Table 1.

## 3. Research Gap and Contribution of This Work

Prior studies have individually addressed components of the rotor-induced measurement bias problem—including rotor wake aerodynamics [9,10,11], UAV-based pollutant sampling [18,19,20], and low-cost sensor calibration [13,14]. But no study integrates turbulence source, sensor error, and correction strategy into a unified, multi-pollutant framework across propeller sizes and rotor configurations (Table 1). Three gaps remain: rotor aerodynamics has not been coupled to quantitative sensor bias estimation; cross-pollutant sensitivity to rotor wake disturbance has not been classified within a single framework; and sensor placement, airflow conditioning, and bias correction have not been consolidated into one analytically grounded tool. Practitioners therefore lack a structured, physics-informed way to estimate uncertainty, choose sensor placement, and plan calibration campaigns before deployment.

This paper addresses that gap. It assembles the analytical chain—from rotor aerodynamics through wake turbulence, sensor response, and measurement bias, to correction strategy—into a single framework that: (i) characterizes and classifies rotor-induced turbulence sources; (ii) defines four sensor contamination zones from momentum-theory wake analysis; (iii) estimates pollutant-specific, speed-dependent measurement bias across nine pollutant species and seven sensor classes, with fully parameterized coefficients for seven classes and provisional bounds for O_3_ and VOCs; (iv) proposes engineering guidelines for sensor placement, airflow conditioning, and bias correction; and (v) provides a structured calibration workflow. All estimates are analytical predictions requiring platform-specific verification. The goal is a physics-constrained foundation for platform design, sensor placement, and calibration planning—not a substitute for experimental calibration.

The novelty lies not in new governing equations, which are established results from the fluid dynamics and aerosol sensing literature, but in integrating these previously disconnected elements into a single analytical methodology, as summarized against prior single-component studies in Table 1. The specific scientific contributions are:

- Systematic integration of established physics-based relations linking rotor-induced airflow disturbance to air quality measurement bias, forming an integrated design and planning tool not previously available in the literature.
- Definition of four UAV sensor contamination zones from rotor wake interaction geometry and momentum-theory wake models, each assigned literature-constrained turbulence-intensity thresholds.
- A pollutant-dependent measurement bias estimation methodology parameterized by flight speed, propeller diameter, and sensor operating principle, covering nine target pollutants across four sensitivity tiers.
- A physics-informed bias correction methodology providing a structured, reproducible approach for future experimental implementation and operational use, supported by a propagated uncertainty analysis that bounds the framework’s defensible accuracy at approximately ±10–15% under representative operating conditions.

## 4. UAV Platforms for Air Pollution Monitoring

Unmanned aerial vehicles (UAVs) are increasingly used for atmospheric sensing and air-quality monitoring because they enable flexible, high-resolution measurements in locations that are difficult to access using conventional monitoring platforms.

UAVs used for atmospheric sensing fall into five broad classes: fixed-wing, hybrid fixed-wing/VTOL, rotary-wing multi-rotor, rotary-wing helicopter, and lighter-than-air [21]. These differ in endurance, flight altitude, maneuverability, payload capacity, and hovering capability. Fixed-wing and hybrid platforms suit long-endurance missions and large-area coverage; rotary-wing and lighter-than-air platforms give greater positional control and hovering capability. Multi-rotor UAVs combine VTOL capability, hovering, low-altitude flight, and high maneuverability, making them well suited to localized air-quality monitoring—slow transects, measurements at predefined locations, and operation in spatially constrained environments. Table 2 compares the main UAV platforms and their characteristics [5].

Despite these advantages, multi-rotor UAVs introduce an important aerodynamic challenge for atmospheric sensing. The rotors that provide hovering and flight control also generate a disturbed flow field around the vehicle. Propeller-induced downwash and blade-tip vortices modify the local airflow. Ambient wind interaction, rotor-to-rotor interference, wake deformation during forward flight, and sensor placement can further affect this flow field. These effects can alter airflow and pollutant transport near the sensor inlet. As a result, the measured concentration may deviate from the true ambient concentration. Characterizing these rotor-induced effects is therefore essential for assessing the accuracy of multi-rotor UAV-based air-quality measurements.

## 5. Mathematical and Conceptual Foundations of Rotor-Induced Turbulence in Air-Pollution Monitoring Using Multi-Rotor UAVs

This section analyzes the turbulence sources generated by multi-rotor UAV platforms and their effect on wake behavior and pollutant transport. It provides the physical basis for linking rotor-induced turbulence to sensor measurement bias.

### 5.1. Scope, Assumptions, and Validation Pathway

This methodology is a physics-informed design and estimation tool. It gives analytical predictions that guide platform design and campaign planning, not regulatory-grade measurements. Hover and constant-speed flight tests (Section 7.1 and Section 7.2) already support the framework’s central Zone 4 prediction and its motion-dependent bias trend. Extending it to more speeds, propeller sizes, and pollutants is the natural next step. To state this scope explicitly: the experimental evidence reported here is limited to a single 21-inch quadrotor platform under hover and one constant-speed (5 m/s) condition, covering five of the nine target pollutants (Section 7.1 and Section 7.2); generalizing the framework’s predictions to other platforms, propeller sizes, flight speeds, and the remaining pollutant classes requires the multi-platform, multi-condition validation campaign outlined above and detailed further in Section 8.3.

The framework rests on five assumptions. (1) Quasi-steady rotor flow: the wake is time-averaged, without resolving unsteady blade-passage or tip-vortex effects; this is reasonable for the mean-field turbulence intensities and bias estimates targeted here, but it does not resolve the blade-passage-frequency pressure spikes noted in Table 3, so instantaneous peak disturbance may exceed the time-averaged predictions used throughout this framework. (2) Incompressible flow: rotor tip Mach numbers stay below 0.3 for most platforms, though the 10-inch and 13-inch propellers in Table 4 reach M ≈ 0.3–0.35 at high RPM, where compressibility affects tip circulation, making those rows more approximate; standard incompressible actuator-disk theory (Equation (1)) applies without correction across most of the platform range examined, and for the higher-Mach rows the predictions should be read as approximate bounds rather than point estimates. (3) A spatially homogeneous ambient pollutant field at the scale of the UAV, reasonable in a well-mixed boundary layer away from strong local sources; near steep concentration gradients, such as plume edges or the stratified layers discussed in Section 5.6.1, this assumption breaks down, and any deviation attributed here to rotor-induced dilution may instead partly reflect real ambient structure. (4) Linear correction superposition: sensor bias scales linearly with turbulence intensity and flow velocity within the operational range tested (hover to 5 m/s); this simplification is consistent with the ±10–15% correction uncertainty reported in Section 5.8, but as noted in Section 8.3 it does not capture non-linear interactions expected at higher speeds or in gusty conditions. (5) Literature-calibrated empirical coefficients, which require platform- and sensor-specific identification before operational deployment, adopted because dedicated multi-platform calibration data are not yet available (Section 8.3); this is reasonable for the design-stage estimates targeted here, but it means the bias magnitudes reported in Section 5, Section 6 and Section 7 carry the ±10–15% propagated uncertainty of Section 5.8 until platform-specific coefficients are measured, and should be read as illustrative rather than universal.

These assumptions set the boundary conditions of the approach and motivate the limitations discussed in Section 8.3.

To make parameter provenance explicit: the electrochemical flow-sensitivity coefficient k_flow_ is adopted from the literature [14] and has not been independently re-measured on the reference platform used in Section 7.1 and Section 7.2; the speed-dependent reduction coefficients α are calibrated against the particulate datasets of [18,19] and the electrochemical gas dataset of [14], and are explicitly marked provisional for the PID/MOS-based VOC tier and undetermined for O_3_ pending wind-tunnel characterization (Section 5.6.2); the turbulence-intensity thresholds defining the contamination zones are constrained by the rotor-wake literature cited in Section 5.2 rather than measured on this platform; and the mast-height placement criterion (Hmast > 3D) follows the same literature-derived wake-decay relations. None of these coefficients has been independently re-calibrated for the specific quadrotor, propeller, and sensor combination used in the present tests; the ±10–15% propagated uncertainty reported in Section 5.8 reflects this literature-to-platform transfer. A dedicated sensitivity analysis quantifying how far predictions would shift under plausible platform-to-platform variation in these coefficients has not been performed here, since generating one without new calibration data would risk manufacturing an unsupported result; this is accordingly listed among the validation priorities in Section 8.3.

**Figure 1:**
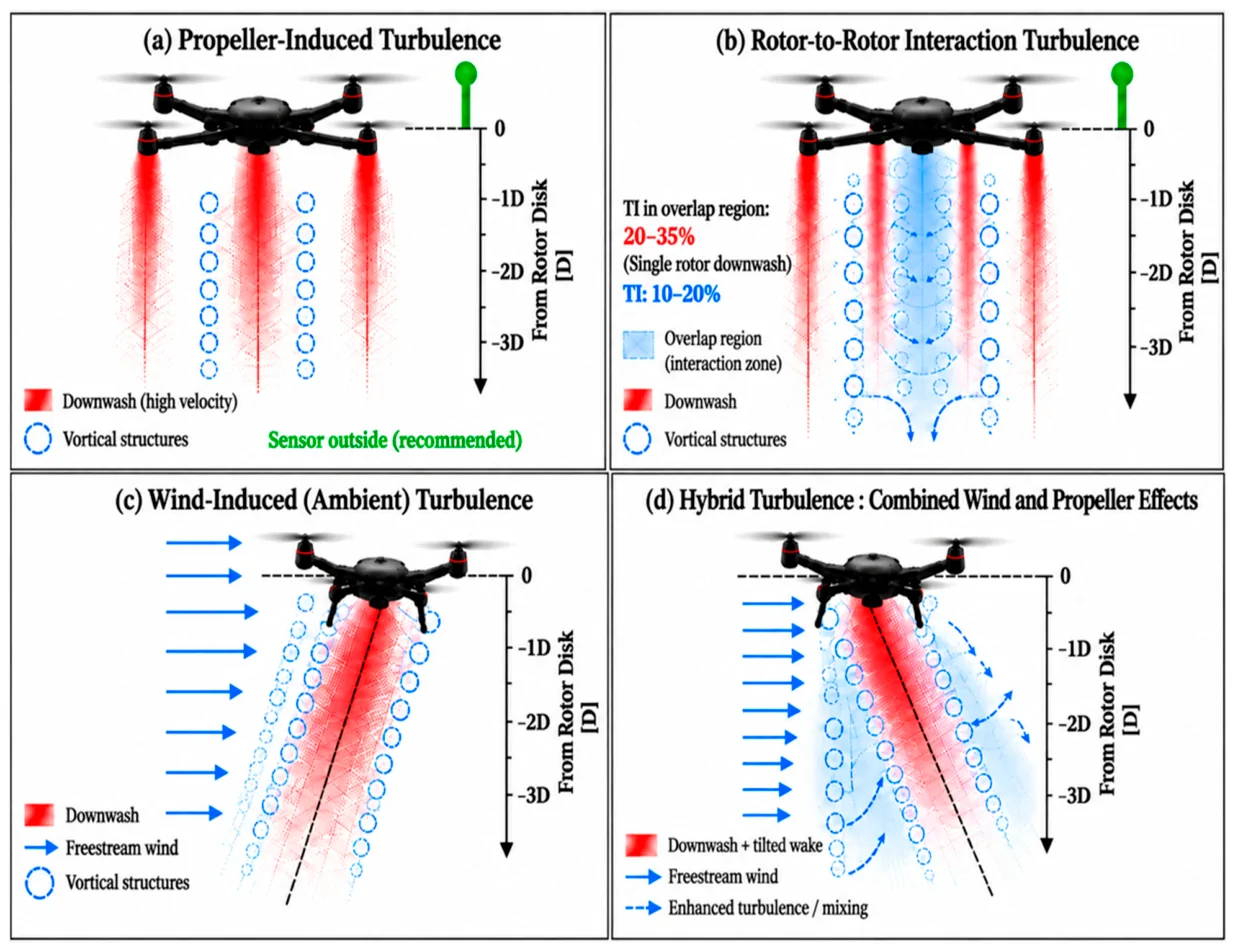
Predicted turbulence sources in multi-rotor UAV operation: (**a**) propeller-induced downwash turbulence; (**b**) rotor-to-rotor interaction turbulence in the inter-rotor overlap region; (**c**) wind-induced (ambient) turbulence; (**d**) hybrid turbulence from combined wind and propeller effects. TI (turbulence intensity) ranges shown are analytical estimates (Section 5.2).

The methodology has three stages. Analytical prediction (Section 5, Section 6 and Section 7) uses physics-constrained models, calibrated against published data, to estimate measurement bias, contamination zone boundaries, and correction factors. Experimental validation adds hover co-location and wind-tunnel tests with grid- or honeycomb-generated turbulence to reproduce realistic intensity levels [22,23], and multi-speed flight campaigns to generate the empirical coefficients needed for the bias correction formulation with quantified uncertainty. Operational deployment follows once these coefficients meet the application’s accuracy needs. The hover and constant-speed tests in Section 7.1 and Section 7.2 complete the first stage; wind-tunnel and multi-speed campaigns are the planned next steps. Figure 1 schematically shows the turbulence-source classification underlying every stage, detailed in the sections that follow.

### 5.2. Definition and Classification of Turbulence Sources

Characterizing multi-rotor UAV turbulence starts with identifying its origin and intensity. Three sources are identified: propeller-induced, wind-induced (ambient), and hybrid. Table 3 classifies these sources by predicted airflow effect and estimated impact on pollutant measurements.

The five scenarios in Table 3 span a wide bias range: 5–20% for ambient wind effects in calm hover, up to 35–55% for worst-case hybrid turbulence in forward flight. No single mitigation strategy covers all conditions.

#### 5.2.1. Propeller-Induced Turbulence

Each rotor in a multi-rotor UAV may be modeled as an actuator disk that accelerates air axially downward to generate thrust. The induced velocity at the rotor disk, v_i_, is obtained from actuator disk momentum theory [24]:

(1) $$v_{i}=\sqrt{\frac{T}{2\rho A}}$$

where ***T*** is rotor thrust [N], ***ρ*** is air density (1.225 kg/m^3^ at sea level, 15 °C), and ***A*** = π · r^2^ is the rotor disk area [m^2^]. By the Rankine–Froude theorem, the slipstream velocity far downstream approaches 2v_i_. For a reference 21-inch quadrotor (total mass 1.5 kg, four rotors), each rotor produces T_r_ = 3.68 N and A = 0.224 m^2^, giving v_i_ = 2.59 m/s. Applying the far-wake limit to the ground plane (V_D_ ≈ 2v_i_) gives a predicted downwash velocity V_D_ of about 5.18 m/s, matching the range reported by [9,10]. This value comes from hover thrust-equilibrium; Table 4’s broader 6.7–8.9 m/s range for the same 21-inch class instead spans the full RPM envelope, so the two figures are not in conflict—Table 4 uses the RPM-range basis, while this section uses the hover-equilibrium value. The downwash jet dilutes local pollutant concentration by displacing ambient air, reducing accuracy for both PM and electrochemical gas sensors in a way that needs platform-specific calibration to quantify precisely.

#### 5.2.2. Rotor-to-Rotor Interaction Turbulence

Adjacent rotors’ wakes interact aerodynamically. Ref. [11] showed close rotor separation produces complex interference affecting thrust and wake structure. Turbulent kinetic energy k in overlapping wake regions is estimated from [25]:

(2) $$k=\left(\frac{3}{2}\right)\cdot {\left(TI\cdot \bar{U}\right)}^{2}$$

where TI is turbulence intensity [-] and $\bar{U}$ is the mean flow velocity [m/s]. In regions where rotor wakes overlap, the framework predicts TI values of 20–35%, compared with 10–20% in a single isolated rotor’s downwash column, consistent with [11]. This amplified turbulence generates non-uniform concentration gradients across the UAV body, requiring position-specific correction.

#### 5.2.3. Wind-Induced (Ambient) Turbulence

**Figure 2:**
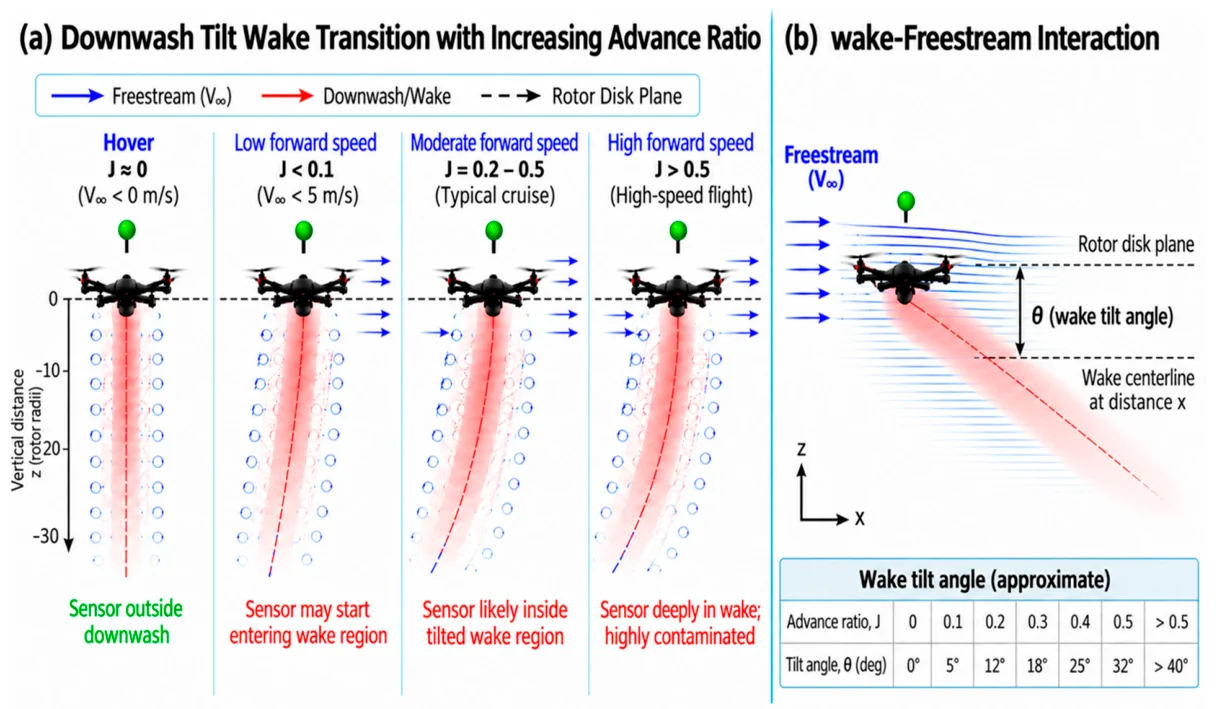
Predicted effect of forward flight speed on rotor wake tilt and sensor contamination risk. (**a**) Wake centerline deflection with increasing flight speed, from vertical downwash in hover to a progressively tilted wake at cruise speed (advance ratio J, Equation (3)). (**b**) Wake tilt angle versus flight speed, computed from the classical relation *χ* ≈ arctan(V∞/v_i_) [24], indicating that sensors mounted behind the rotor plane are predicted to enter the contaminated wake during forward flight.

Ambient wind interacts with the UAV airframe and propeller wakes, adding a turbulence component atop the rotor-induced baseline. The advance ratio J characterizes wake tilt as a function of flight or wind speed:

(3) $$J=\frac{V_{\infty }}{n\cdot D}$$

where V_inf_ is the freestream or wind speed [m/s], ***n*** is rotor rotational speed [rev/s], and ***D*** is rotor diameter [m]. At J > 0.2, the framework predicts significant wake tilt: sensors outside the calm-hover contamination zones may enter the turbulent wake envelope during crosswind operations. Wind-induced turbulence alone contributes an estimated 5–15% TI increment at the sensor, consistent with [26,27]. J is used throughout as the sole parameter governing wake tilt—a first-order approximation for a fixed thrust axis. In practice, wake skew also depends on pitch attitude and disk loading, which J alone does not capture [24]. The tilt predictions in Figure 2 and Section 5.5.3 should therefore be read as conservative estimates.

#### 5.2.4. Hybrid Turbulence: Combined Wind and Propeller Effects

During forward flight, propeller-induced downwash and the ambient freestream combine into the most complex disturbance field in UAV-based monitoring—the worst case for sensor accuracy. The mean pollutant transport equation, including the turbulent diffusion term, is:

(4) $$\frac{𝝏C}{𝝏t}+\bar{u}\cdot 𝛁C=𝛁\cdot \left(D_{eff}\cdot 𝛁C\right)+S_{c}$$

where ***C*** is mean pollutant concentration [mol/m^3^ or µg/m^3^], D_eff_ = D_mol_ + D_turb_ is the effective diffusivity [m^2^/s], and S_c_ represents chemical source or sink terms. Turbulent diffusivity D_turb_ is approximated here by the standard k-ε eddy-viscosity relation [25], which strictly gives the turbulent (eddy) viscosity ν_t_; D_turb_ = ν_t is adopted by implicitly assuming a turbulent Schmidt number Sc_t_ ≈ 1:

(5) $$D_{\mathrm{turb}}=\frac{C_{\mu }\cdot k^{2}}{\varepsilon },C_{\mu }=0.09$$

Elevated D_turb_ in hybrid turbulence regions is predicted to speed lateral dispersion of pollutant plumes, diluting sensor-inlet concentrations beyond what either mechanism produces alone. Equations (4) and (5) are qualitative context, not operational components: ε is not estimated here, so D_turb_ remains indeterminate and no table value is computed from them. Cμ = 0.09 is the standard k-ε constant for flat-plate shear flow [25]; its validity for a rotor wake’s curved, rotating flow is unverified [28], adding a model-form uncertainty not included in the Section 5.8 budget.

### 5.3. Turbulence Intensity and Wake Structure

Turbulence intensity (TI) is formally defined as [25]:

(6) $$TI=\frac{\sigma_{u}}{\bar{U}}$$

where σ_u_ is the standard deviation of streamwise velocity fluctuations [m/s] and $\bar{U}$ is the time-averaged mean velocity [m/s]. Based on the experimental hot-wire anemometry data of [9] and the CFD characterization of [10], TI is estimated to peak at 25–40% in the blade-tip vortex annulus, drop to 15–25% in the central downwash column (Zone 1), and recover to ambient levels (TI < 5%) beyond 3–5 rotor diameters above the rotor plane—a lower-turbulence region governed by disk inflow and airframe recirculation rather than the downwash wake. These thresholds come mainly from Shouji et al.’s [9] single-rotor agricultural drone data, whose geometry and RPM differ from ours. Because tip vortex strength scales with blade Reynolds number [29], the 25–40% Zone 2 TI range should be treated as a first-order estimate pending dedicated multi-rotor characterization. For spatial consistency, this worked example pairs the Zone 1 axial downwash velocity with the Zone 1 TI range (Table 5): for the reference 21-inch quadrotor case with V_D_ = 5.18 m/s and TI = 0.20 (the upper bound of the 15–25% Zone 1 range):

(7) $$k=\left(\frac{3}{2}\right)\cdot {\left(0.20\cdot 5.18\right)}^{2}=1.61\;m^{2}/s^{2}$$

This elevated kinetic energy drives rapid mixing of nearby plumes, biasing sensor readings toward a diluted, spatially averaged concentration rather than the true near-field value. The estimate assumes locally isotropic turbulence (k = (3/2)(TI·V)^2^); since the rotor near-wake is markedly anisotropic, it is an order-of-magnitude indicator rather than a precise value, pending anisotropic corrections from the planned wind-tunnel tests.

### 5.4. Effect of Propeller Size on Turbulence and Measurement Accuracy

**Figure 3:**
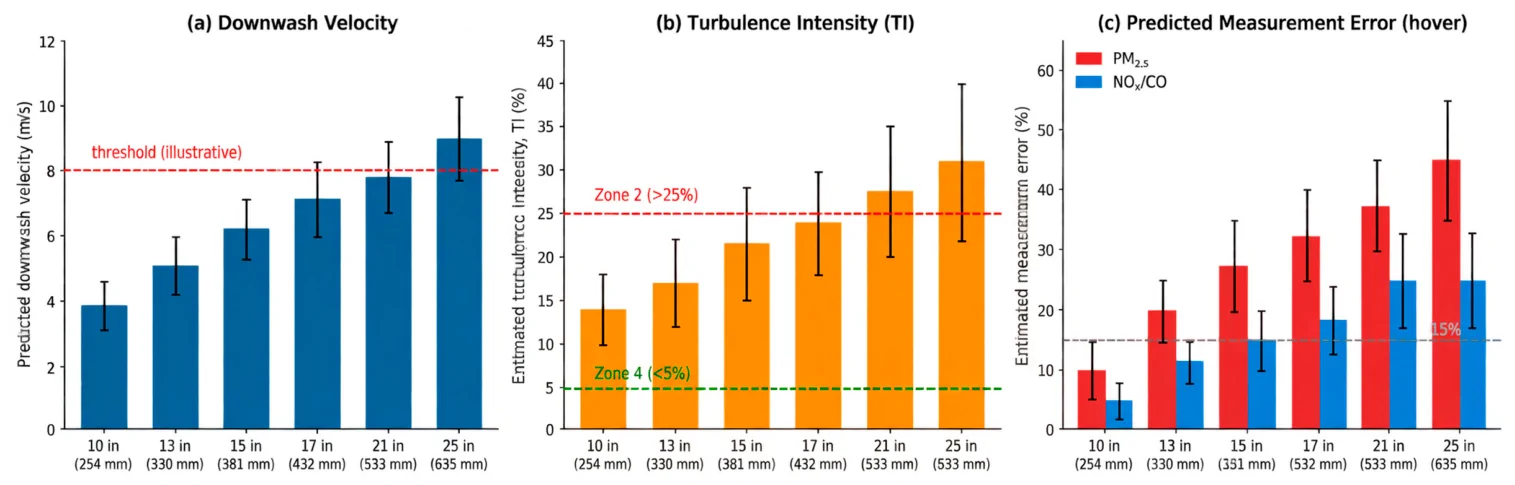
Analytically predicted effect of propeller diameter on downwash velocity (**a**), peak turbulence intensity (**b**), and hover-mode measurement bias for PM and gas sensors (**c**), derived from Equations (1)–(7) and constrained by the experimental datasets of Kartal et al. [10] and Shouji et al. [9]. Horizontal dashed lines indicate the Zone 4 TI threshold (<5%) and Zone 2 boundary.

Propeller diameter is one of the most influential design parameters governing the predicted turbulence intensity and its estimated effect on sensor accuracy. The downwash velocity and airflow volume estimates in Table 4 derive from Equation (1) across manufacturer-reported RPM ranges, constrained by the experimental data of [9,10]. The “Est. TI—central column” values represent the central downwash column (Zone 1), distinct from the higher blade-tip-vortex-annulus values (Zone 2, 25–40%) in Table 5 and Section 5.3. Table 5’s Zone 1 range (15–25%) is a general cross-platform band, while Table 4’s values are platform- and RPM-specific and, for the 21-inch and 25-inch configurations, extend above it (20–35%, 22–40%). For the reference quadrotor, the Table 4 value is the applicable bound where the two diverge. The resulting trends are summarized in Figure 3.

Table 4 shows a consistent trend: as propeller diameter increases, downwash velocity, airflow volume, and turbulence intensity all rise, increasing predicted measurement bias. Platforms with 10–13-inch propellers are estimated to keep PM bias below 25% with careful sensor placement; 21–25-inch propellers need both elevated mast mounting (Section 6.2) and bias correction (Section 5.9) for good measurement performance.

**Figure 4:**
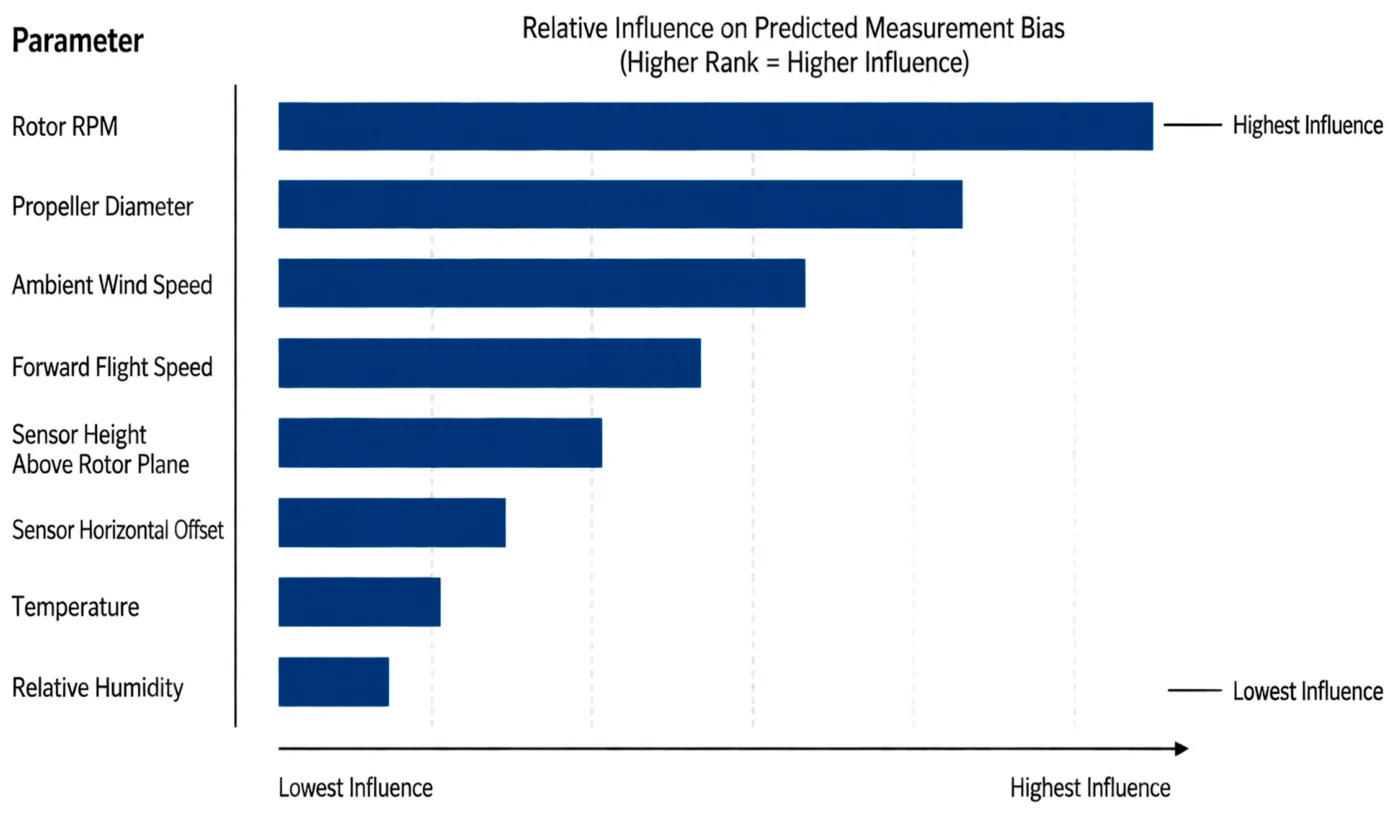
Conceptual sensitivity ranking of the governing parameters influencing predicted UAV rotor-induced measurement bias, based on the analytical formulation presented in this study.

The framework’s parameters contribute unequally to predicted measurement bias. Figure 4 gives a conceptual sensitivity ranking of the main model inputs, meant to aid interpretation rather than serve as a platform-specific quantitative sensitivity analysis.

Rotor RPM and propeller diameter dominate, since they set the strength of the rotor-induced flow field; wind speed and forward flight speed modify wake transport, sensor placement governs the local sampling environment, and ambient temperature and humidity play a minor role. Quantitative global sensitivity indices would need platform-specific calibration and are beyond this study’s scope.

### 5.5. Airflow Behavior and Sensor Contamination Zone Classification

This section maps the spatial distribution of the UAV-induced airflow field into contamination regions of differing aerodynamic disturbance, which in turn support sensor placement recommendations.

#### 5.5.1. Multi-Rotor Airflow Structure

The model-derived airflow field around a hovering multi-rotor UAV has three components: (1) the downwash column, a jet of accelerated air directed vertically downward beneath each rotor disk; (2) the blade tip vortex sheet, a helical structure shed from each blade tip that carries intense turbulent energy radially outward; and (3) the induced lateral flow, generated by the rotor pressure field above and beside the disk plane. In forward flight, the advance ratio J (Equation (3)) governs how these structures tilt and merge, potentially engulfing sensor positions that stayed clean during hover. For pollutant measurement, local airflow velocity and turbulence intensity set the transport pathway from ambient air to the sensor inlet.

#### 5.5.2. Contamination Zone Classification

**Figure 5:**
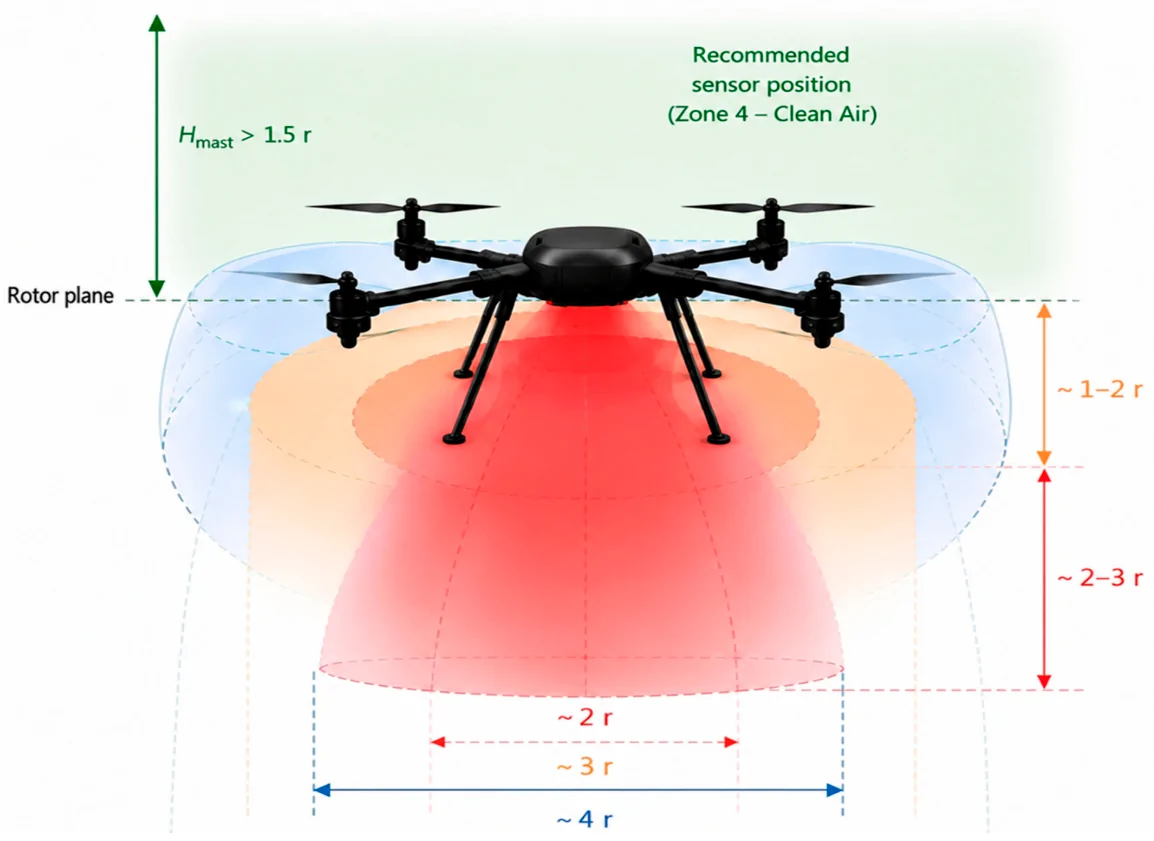
Predicted contamination zone boundaries around a multi-rotor UAV (conceptual representation based on the analytical framework developed in this study): downwash zone (Zone 1), blade tip vortex annulus (Zone 2), lateral induced flow zone (Zone 3), and the low-disturbance air region above the rotor plane (Zone 4), recommended for sensor placement. The boundaries of these zones can be adapted to the aerodynamic characteristics of individual UAV platforms and further refined through platform-specific measurements for operational applications.

Four analytically distinct sensor contamination zones are identified around multi-rotor UAVs based on rotor wake interaction geometry, synthesizing the experimental wake data of [9,11,18] within a momentum-theory spatial framework (Equations (1)–(7)), constrained by the sensor placement studies of Wang et al. [11] and Pochwala et al. [12]. Table 5 presents the full classification. The corresponding spatial zone geometry is illustrated in Figure 5.

#### 5.5.3. Wake Tilt During Forward Flight

In forward flight, the downwash column tilts progressively rearward as the advance ratio J increases. Tilt angles in Figure 2 come from the classical wake skew relation for level flight, *χ* ≈ arctan(V∞/v_i_) [24], applied as a first-order approximation for the multi-rotor case (limitations in Section 5.2.3). This single-rotor wake-skew relation also does not represent aerodynamic interaction between adjacent rotors on the same frame, nor differential thrust across rotors during maneuvering (e.g., coordinated turns or rapid climbs/descents), both of which can shift the wake envelope relative to the symmetric-thrust, steady-level-flight regime assumed here; the resulting predictions should accordingly be read as applicable to steady, level, symmetric-thrust forward flight, with maneuvering conditions identified as an open extension for future work. At J < 0.1 (near-hover), the downwash column stays roughly vertical; at J = 0.2–0.5 (10–20 km/h cruise), significant wake tilt is predicted, and sensors aft of the center of gravity may enter the turbulent wake even if clean during hover. This tilt underlies the speed-dependent bias correction model in Section 5.7; Section 5.6 quantifies how these sources translate into pollutant-specific bias across the operational speed range.

#### 5.5.4. Vertical Motion During Hover

The zone-boundary analysis above (Section 5.5.1, Section 5.5.2 and Section 5.5.3) treats sensor altitude as fixed, implicitly assuming exact hover. In practice, a hovering multi-rotor platform does not hold altitude perfectly: barometric- and GNSS-based altitude-hold controllers admit residual vertical velocity and short-term altitude excursions around the commanded setpoint. Because the mast-height criterion described in Section 6.2 (H_mast > 3D) defines a single static boundary between the turbulent near-wake and the low-disturbance Zone 4, a vertical excursion large enough to move the sensor below this boundary would transiently reintroduce it into Zone 1–2 turbulence even while the platform is nominally hovering at the target altitude above the rotor plane. This mechanism is distinct from the forward-flight wake tilt addressed in Section 5.5.3, since it can occur at zero advance ratio (J ≈ 0).

Altitude estimation on small multi-rotor platforms typically combines GNSS and barometric measurements. GNSS vertical-position solutions are well established in the GNSS literature to carry substantially larger uncertainty than horizontal position, owing to satellite-geometry effects, and barometric altitude is itself sensitive to the local pressure field that the rotors’ own downwash perturbs. Both effects act in the same direction: they reduce confidence in exactly how far above the rotor plane the sensor sits at a given instant, which is precisely the quantity the static mast-height boundary assumes is well controlled. This effect is discussed further as a limitation of the present validation in Section 8.3.

### 5.6. Effects of Turbulence and Airflow on Pollutant Measurement

The contamination zones identified above directly affect pollutant measurement accuracy. This section covers how rotor-induced airflow affects sensor performance: the mechanisms behind systematic bias, how different pollutant classes respond, and the resulting concentration deviations across flight conditions. The chain linking rotor operation to measurement bias proceeds in two coupled stages, using only the quantities already introduced above. First, rotor thrust and RPM set the induced and downwash velocity (Equation (1)), which combine with the advance ratio J (Equation (3)) to determine the turbulent kinetic energy and turbulence intensity TI (Equations (2), (6) and (7)); these TI values in turn define the contamination-zone boundaries (Table 3 and Table 5) that govern where a sensor can be placed to avoid the most disturbed flow. Second, within a given placement zone, the local flow velocity actually reaching the sensor inlet—denoted V_flow_ in the electrochemical response model (Equation (8)) and normalized flight speed v in the motion-correction model (Equation (9))—is itself set by the same rotor-induced velocity field and advance ratio that generated the zone’s TI, so it is not an independent input but a direct consequence of the rotor characteristics traced in Section 5.2 and Section 5.3. TI therefore governs which zone a sensor occupies and hence its exposure regime, while V_flow_ and v govern the quantitative magnitude of bias predicted within that regime via Equations (8) and (9). A single closed-form expression giving bias directly as a function of TI has not been fitted here, because the calibration datasets underlying Equations (8) and (9) report bias against flow velocity and flight speed rather than against TI directly; deriving that direct TI-to-bias mapping is accordingly identified as a target for the planned wind-tunnel campaign (Section 5.1) rather than asserted here without supporting data.

The quantitative estimates in this section are analytical predictions from models calibrated using previously published experimental datasets [9,13,14,15,18,19,20], reflecting model-based trends rather than direct measurements from a specific platform; the reported (±) ranges reflect variability among the referenced datasets, not the framework’s propagated uncertainty (Section 5.8).

#### 5.6.1. Physical Mechanisms of Measurement Error

Electrochemical sensors, widely deployed in low-cost air quality networks [30], operate on a diffusion- or flow-limited mass transfer principle. At elevated airflow velocities, as in propeller downwash or forward flight, convective mass transfer dominates over molecular diffusion and analyte contact time at the sensing electrode decreases. Measured concentration C_meas_ relative to true ambient concentration C_true_ is modeled as:

(8) $$\frac{C_{\mathrm{meas}}}{C_{\mathrm{true}}}=1-k_{\mathrm{flow}}\cdot \sqrt{V_{\mathrm{flow}}}$$

Fine particles (PM_1_, PM_2.5_) with Stokes number St ≪ 1 are predicted to follow airflow streamlines closely, so OPC-measured particle number concentration drops in proportion to sensor sampling flow relative to freestream entrainment. Coarser particles (PM_10_, St ≈ 1, evaluated at the blade-tip-vortex core scale) resist deflection more but still deviate in the tip vortex region—an isokinetic bias distinct from OPC coincidence error (undercounting from simultaneous particles in the sensing volume)—consistent with Vincent [31] and Belyaev and Levin [32], while coincidence error is discussed separately in Hinds [33].

In a stably stratified boundary layer, UAV-induced turbulence is expected to mix and homogenize vertical concentration gradients near the platform—most pronounced when the rotor-generated turbulent kinetic energy is enough to overcome buoyant suppression of vertical motion, i.e., when the local gradient Richardson number Ri = (g/θ)(∂θ/∂z)/(∂U/∂z)^2^ drops below its critical value (Ri_cr_ ≈ 0.25) under the added rotor-induced shear [34]. The sensor then samples a vertically averaged rather than point concentration, introducing altitude-dependent bias in vertical profiling—an effect documented empirically by Ahlawat et al. [7] in an urban vertical profiling study. Because this mixing destroys the gradient before it reaches the sensor, it is irreversible information loss rather than a correctable bias: Equation (10) cannot reconstruct vertical structure already lost. Vertical-gradient mapping is therefore best treated as valid mainly under low-wind, stably stratified conditions (Ri > Ri_cr_), and with caution otherwise.

#### 5.6.2. Pollutant-Specific Sensitivity Classification

Each pollutant class responds differently to rotor turbulence, owing to its physical/chemical properties and its sensor’s operating principle. Table 6 presents a four-tier sensitivity classification based on the mechanisms above, constrained by experimental evidence from [13,14,15].

The flow-sensitivity coefficients above are specific to each sensor’s operating principle and not interchangeable. Electrochemical sensors (NO_2_, EC-variant CO) are diffusion/flow-limited, so convective mass transfer gates analyte contact time—modeled by k_flow_ (units (m/s)^−0.5^, so k_flow_·√V_flow_ is dimensionless) in Equation (8), consistent with Spinelle et al. [14]. NDIR sensors (CO, CO_2_) sample via a flow-through cell and are comparatively flow-insensitive. UV photometric O_3_ sensors rely on Beer–Lambert absorption rather than surface delivery, so the electrochemical model does not apply; its coefficient is undetermined pending wind-tunnel data. Within the VOC tier, PID and MOS sensors are both flow-sensitive but via different mechanisms, so the shared α coefficient in Table 7 is provisional. The tier assignments in Table 6 group pollutants primarily by this shared response mechanism and its associated correction coefficient, not by directly comparing the qualitative sensitivity columns across mechanisms; those columns describe relative severity within a given sensor’s own response model rather than an absolute scale common to all sensor types. This is why NH_3_, measured with the electrochemical, k_flow_-governed sensors described above, is grouped in Tier II alongside NO_2_ and CO despite a “Medium” rating, while VOCs measured by PID/MOS, which follow the separate, provisional α coefficient, are grouped in Tier III: the tier reflects which correction model and evidence base applies, not a claim that NH_3_ underlying sensitivity is categorically lower than that of VOCs.

### 5.7. Concentration Reduction as a Function of Flight Speed

The fractional reduction in measured concentration relative to hover conditions is modeled as a linear function of normalized forward flight speed:

(9) $$f_{\mathrm{motion}}\left(v\right)=\alpha \cdot \frac{v}{v_{\max}}$$

where *α* is the pollutant-class-specific maximum reduction fraction at the reference speed v_max_ = 16.7 m/s (60 km/h). This linear fit does not encode the wake-tilt transition (Section 5.5.3: near-zero tilt below J ≈ 0.1); it likely overstates the correction at low speeds and is most reliable in the mid-to-high speed range spanned by the calibration data. The α coefficients are calibrated against the experimental datasets of [18,19] for particulate species, and from [14] for electrochemical gas sensors. This is an empirical fit to the aggregate speed-bias trend, distinct from the √V_flow_ scaling used for k_flow_ in Equation (8), which models the electrochemical mass-transfer mechanism specifically—so α and k_flow_ should not be treated as interchangeable without dedicated calibration. The corrected concentration C_corr_, from the full two-stage formulation, is presented in Section 5.9 (Equation (10)).

f_env_ is the environmental correction factor for wind speed, temperature, and relative humidity, defined in Section 5.9 (Equation (11)). Table 7 gives estimated concentration reductions across flight speeds for each pollutant class. Equation (9)’s linear form is calibrated directly against the discrete-speed datasets of [18,19] for particulates. It does not match the square-root mass-transfer dependence used for electrochemical sensors in Equation (8), since available data cannot constrain an independent V^0.5^ fit; the two forms should not be combined for the same sensor without reconciliation, and platform-specific calibration (Section 5.1) is recommended before either is applied outside its calibration range. This model-form uncertainty is not included in the Table 7 ranges, which reflect only coefficient (α) uncertainty.

### 5.8. Measurement Uncertainty in Relation to Turbulence

The principal sources of uncertainty in the proposed methodology are characterized below to establish its defensible bounds, following the Guide to the Expression of Uncertainty in Measurement (GUM) approach [35,36].

Model coefficient uncertainty: the empirical coefficients used throughout—α (Equation (9)), k_flow_ (Equation (8)), k_W_, k_T_, k_RH_ (Equation (11)), and Cμ (Equation (5))—are sourced from the literature, not platform-specific measurement. The α coefficient for PM sensors spans 0.33–0.57 (±0.12), and k_flow_ for electrochemical NO_2_ sensors spans roughly a factor of four across commercial designs; both need sensor-specific wind-tunnel calibration to resolve.

Turbulence intensity estimation uncertainty: TI values in Table 3, Table 4 and Table 5 derive from Equations (2), (6), and (7), calibrated against the experimental ranges of Shouji et al. and Rai et al. Actual platform-specific values may deviate by ±5–10 points depending on rotor geometry, RPM, and operating conditions.

Correction factor uncertainty: f_env_ (Equation (11)) depends on three sensitivity coefficients (k_W_, k_T_, k_RH_) spanning roughly a factor of three across the literature; propagated uncertainty in f_env_ under representative field conditions is approximately ±8–15%.

Propagated combined uncertainty: treating each coefficient as a Type B, rectangular uncertainty and combining the motion, flow, and environmental terms in quadrature at a representative speed (30 km/h) under moderate conditions gives an expanded uncertainty (k = 2, ~95%) of about ±10–15% for PM sensors and ±8–13% for electrochemical gas sensors—adequate for pre-deployment planning but not for regulatory grade monitoring without platform-specific calibration (Section 5.1). The ±8–13% gas sensor bound applies to the narrower, sensor-model-specific k_flow_ sub interval, not the full factor of four span quoted above for commercial NO_2_ designs generally: across the full [0.05, 0.20] (m/s)^−0.5^ range, u(k_flow_) ≈ 0.043 (m/s)^−0.5^ alone contributes u(C_meas_/C_true_) ≈ 9.8% at V_D_ = 5.18 m/s, rising toward ~20% at the upper end—exceeding the declared bound on its own. Outside a calibrated, sensor-specific k_flow_ range, ~20% should be treated as the applicable expanded uncertainty until wind-tunnel calibration narrows it. This propagation is a pre-calibration planning estimate, not a full metrological budget: it assumes coefficient independence, though some (e.g., k_W_ and α) are physically coupled; assumes rectangular distributions where the true shape is unknown; excludes model-form uncertainty from the functional forms of Equations (8)–(11) themselves, which would widen the bounds further; and assumes approximate normality for the k = 2 coverage factor, unverified for this coefficient set. A full GUM-compliant uncertainty budget is a priority for the planned calibration campaign (Section 5.1).

Empirical cross-check: these propagated bounds are broadly supported by the two flight tests on the reference quadrotor (Section 7). The bias figures compared here are time-averaged means, while the propagated k = 2 uncertainty formally bounds individual measurement residuals, which scatter more widely than the mean—so a mean bias within the envelope is a necessary but not sufficient check, as the lower correlations and non-trivial RMSE in Tables 11 and 12 suggest. In the near-zero-motion hover test, observed bias (3.1–9.7%) fell comfortably within the propagated envelope. In the 5 m/s constant-speed test, observed mean bias (6.3–14.9%) stayed within the envelope for four of five pollutants, with PM_10_ marginally exceeding the PM bound. But a mean-bias comparison understates the applicable uncertainty, which should include the random (scatter) component: decomposing RMSE into systematic and random parts (RMSE^2^ = bias^2^ + random^2^) at 5 m/s gives empirical expanded (k = 2) uncertainties of ≈±25% (CO), ±21% (NO_2_), ±25% (O_3_), ±32% (PM_2.5_), and ±34% (PM_10_)—all exceeding the declared ±10–15% (PM)/±8–13% (gas) bounds. The declared bounds appear to capture the systematic component reasonably but understate total empirical uncertainty at this speed, motivating the planned calibration campaign rather than treating current bounds as validated for forward flight. These two data points (hover and a single 5 m/s condition) corroborate the propagated envelope at those speeds, but with only two conditions cannot distinguish a linear speed-dependence from a power-law or piecewise relationship (e.g., the wake-tilt transition near J ≈ 0.2–0.5, Section 5.5.3); at least three non-zero-speed points would be needed to test linearity, motivating the planned multi-speed campaign. Tables 11 and 12 report raw, uncorrected bias, not Equation (10) output, so they are unaffected by the Section 5.9 reordering; verifying Equation (10)’s post-correction accuracy empirically remains a task for the planned calibration campaign.

### 5.9. Bias Correction Formulation

The analytically derived corrected pollutant concentration C_corr_ is obtained through a two-stage multiplicative correction that accounts for motion-induced and environmental bias factors:

(10) $$C_{\mathrm{corr}}=\frac{\left(C_{\mathrm{meas}}\right)}{\left(1-f_{\mathrm{motion}}(v)\right)\cdot f_{\mathrm{env}}(W,T,RH)}-k_{\mathrm{base}}$$

The two correction stages are applied in sequence, not conflated: the multiplicative aerodynamic dilution correction is applied first, then the additive k_base_ offset (Section 6.3.1) is subtracted. This ordering matters because the two errors are mechanistically distinct—dilution scales the whole signal, while k_base_ is a fixed zero-offset that should not itself be scaled by the aerodynamic denominator; subtracting it before division would let a fixed offset appear as a disproportionately large fractional correction whenever concentration departs from the hover-calibration level. Under hover conditions (f_motion_ = 0, f_env_ = 1), Equation (10) still reduces to C_corr_ = C_meas_ − k_base_, so the hover results in Section 7.1 remain valid; only the forward-flight correction (Section 7.2) is affected by the reordering. The equation is defined only where the denominator (1 − f_motion_(v))·f_env_(W,T,RH) stays positive, and k_base_’s weight grows with the aerodynamic correction factor at high speed; practitioners should flag negative or undefined results as invalid rather than clip them to zero. The motion term f_motion_(v) follows Equation (9) with pollutant-class-specific α coefficients (Table 7). f_env_ accounts for ambient wind speed W [m/s], temperature T [°C], and relative humidity RH [%]:

(11) $$f_{\mathrm{env}}=1+k_{W}W+k_{T}\left(T-T_{\mathrm{ref}}\right)+k_{RH}\left(RH-RH_{\mathrm{ref}}\right)+k_{P}\frac{P-P_{\mathrm{ref}}}{P_{\mathrm{ref}}}$$

where k_W_ = 0.01–0.03 (m/s)^−1^, k_T_ = −0.002 to −0.008 °C^−1^ (of the expected sign per standard heat- and mass-transfer scaling [37]), and k_RH_ = 0.001–0.005%^−1^ are indicative ranges derived from [14,19]. Unlike f_motion, f_env is not a pure dilution discount but a net sensor-response modifier: for PM sensors, wind is expected to dilute the sampled plume, while for electrochemical gas sensors, wind can instead enhance analyte delivery via boundary-layer mass transfer—opposite effects that a single, sensor-class-independent k_W_ oversimplifies, so calibration should verify k_W_’s sign separately for each sensor class. The coupling between f_motion and f_env is not yet independently verified and is a priority for the wind-tunnel calibration in Section 5.1. The pressure/altitude term uses P as ambient pressure [k_Pa_] and P_ref_ = 101.325 k_Pa_ (sea level);

k_P_ remains undetermined pending experimental characterization. These coefficients come from qualitative literature sensitivity information, so Equation (11) cannot be applied predictively without prior calibration (Section 5.1); until then it should use k_P_ = 0, valid only near the calibration altitude. The motion term dominates uncertainty in Equation (10) at intermediate speeds (10–40 km/h), underscoring the importance of multi-speed calibration.

### 5.10. Analytical Predictions: Contamination Zones and Pollutant Bias Estimates

This section presents the analytical, model-based outputs obtained by applying the framework of Section 5 to the reference platform: predicted sensor contamination zone characteristics, pollutant-specific sensitivity tiers, and speed-dependent concentration reduction. These are theoretical predictions rather than measurements; the experimental results that test them follow in Section 7.1, Section 7.2 and Section 7.3.

Table 5 provides a structured basis for sensor placement decisions. Zone 1 (primary downwash) and Zone 2 (blade tip vortex annulus) are predicted unsuitable for all pollutant sensor types, with estimated bias of 30–60% and 15–40%, respectively. Zone 4 (above the rotor plane, H_mast_ > 3D) is estimated to give near-ambient conditions (TI < 5%), consistent with the experimental findings of Brosy et al. [20] and Wang et al. [18]. The Zone 2b (inter-rotor) turbulence-intensity range comes from formation-flight wake-interaction studies between separate aircraft rather than co-mounted, rigidly coupled rotors on a single multi-rotor frame, so it should be treated as an order-of-magnitude proxy pending platform-specific characterization.

**Table 6:** Pollutant sensitivity to multi-rotor UAV turbulence sources: four-tier classification based on sensor operating principle and experimental constraints from [13,14,15]. Tier II (electrochemical) bias ranges span roughly a factor of four across the k_flow_ coefficient (Equation (8), Section 5.6.1); read as indicating meaningful effect rather than a quantitative threshold, until wind-tunnel calibration narrows k_flow_.

Pollutant	Sensor Type	Sens. to Rotor TI	Sens. to Downwash	Sens. to Hybrid	Sens. to Fwd Speed	Tier	Notes
PM_1_	Optical/Laser OPC	Very High	Very High	Very High	Very High	I	Strong effect; hover preferred; trajectory deflection dominant
PM_2.5_	Optical/Laser OPC	Very High	Very High	Very High	Very High	I	PM1 like; inertia and dilution dominant.
PM_10_	Optical/Laser OPC	High	High	High	High	I–II	Larger particles partly resist deflection; Zone 2 trajectory bias remains
NO_2_	Electrochemical	High	High	High	High	II	Flow sensitive; contact time decreases at high velocity
NH_3_	Electrochemical	Medium	Medium	Medium	Medium	II	Flow sensitive; Tier II pending sensor specific characterization
O_3_	UV Photometric	High	High	High	High	II	O3 entrainment may cause positive bias, while NO titration may partly offset it [38]
CO (EC)	Electrochemical	High	Medium	High	High	II	Flow sensitive; contact time decreases at high velocity, as for NO2
CO (NDIR)	NDIR	Low	Low	Low	Low–Med	IV	Flow through design; relatively robust to flow effects; least affected CO variant
VOCs (PID)	Photoionization	Medium	Medium	Medium	Medium–High	III	Flow sensitive; provisional α pending sensor specific characterization
VOCs (MOS)	Metal-oxide semiconductor	Medium	Medium	Medium	Low	III	Low flow sensitivity; humidity and temperature cross sensitivity dominate
CO_2_	NDIR	Low	Low	Low	Low–Med	IV	NDIR flow-through design most robust to flow perturbation; predicted least affected

Table 6’s tier assignments carry practical implications: Tier I pollutants (PM_1_, PM_2.5_, PM_10_) are predicted most severely affected, since optical particle counters need near-laminar, isokinetic flow and any turbulence-induced trajectory deviation or coincidence event directly corrupts the count [13,15], supporting hover-only sampling for Tier I on large-rotor platforms. (True coincidence error is governed by ambient particle concentration and flow rate, not rotor turbulence intensity, so it is not itself a rotor-specific artifact—the dominant rotor-wake mechanism for Tier I sensors is the isokinetic trajectory deviation described above.) Tier IV (CO_2_) is estimated to be most robust, since higher molecular weight limits turbulent diffusion losses and NDIR designs tolerate variable flow.

**Table 7:** Estimated concentration reduction (%) by flight speed and pollutant class, calculated from Equation (9); uncertainty ranges reflect literature spread [13,14,15,19].

Pollutant Class	Hover (0 km/h)	10 km/h	20 km/h	40 km/h	60 km/h	Primary Error Mechanism	Max α Coeff.
PM1/PM2.5/PM10	0% ± 3%	8% ± 5%	15% ± 6%	30% ± 9%	45% ± 12%	Particle inertia and turbulent dilution (Equations (1) and (9)); α = 0.45 is a conservative upper bound based on Wang et al. [18], Rai et al. [13], and Peters et al. [15]	0.45 ± 0.12
CO/NO_2_ NH_3_	0% ± 2%	6% ± 4%	12% ± 5%	23% ± 7%	35% ± 10%	Electrochemical and NDIR flow sensitivity (Equation (8), Spinelle et al. [14]); not applicable to UV photometric O3	0.35 ± 0.10
VOCs (PID/MOS)	0% ± 2%	6% ± 4%	12% ± 5%	23% ± 7%	35% ± 10%	Reduced contact time and humidity sensitivity [14]; PID and MOS require separate calibration	0.35 ± 0.10
CO_2_	0% ± 1%	3% ± 2%	7% ± 3%	13% ± 4%	20% ± 6%	NDIR flow-through sensing is inherently robust to external flow perturbation.	0.20 ± 0.06

Table 7 shows PM sensors have the steepest speed-dependence, with bias rising from below 3% in hover to 45% ± 12% at 60 km/h—favoring hover-and-sample protocols for PM_2.5_ on large-rotor platforms. Tier IV pollutants stay at or below 20% bias across the full speed range, and all classes approach near-zero bias at hover, reinforcing hover-and-sample with elevated mast placement as the best overall configuration. The wide uncertainty ranges (typically ±5–12%) reflect genuine variability across the referenced datasets, underscoring the need for platform-specific calibration before operational use.

## 6. Materials and Methods

### 6.1. Reference UAV Platform and Sensor Suite

**Figure 6:**
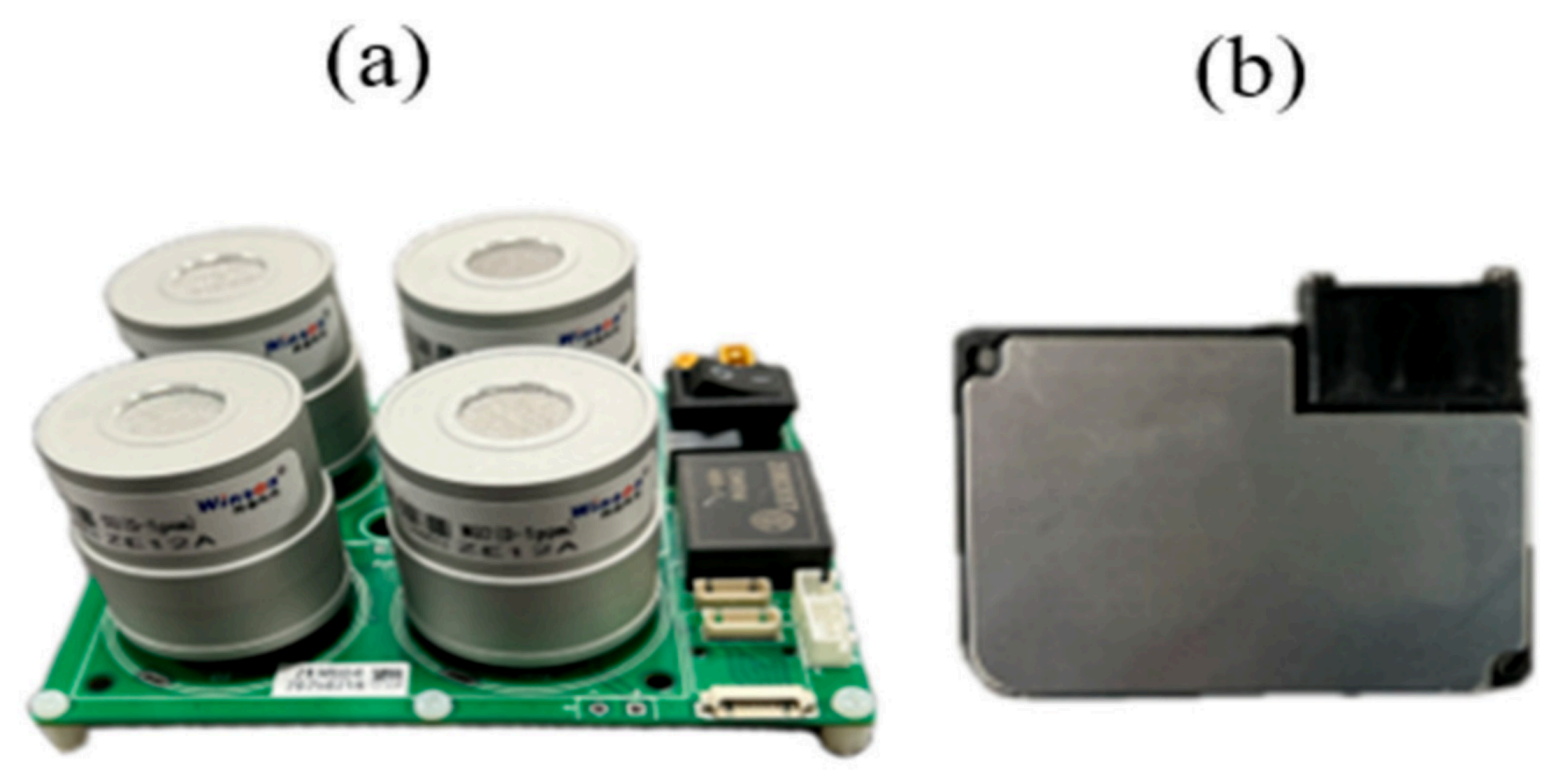
Gas sensor suite integrated into the UAV monitoring system: (**a**) ZEHS04 multi-in-one module housing CO, NO_2_, and O_3_ electrochemical sensors (ZE12A series); (**b**) ZH03B optical particulate matter sensor (PM_2.5_/PM_10_) [40].

**Figure 7:**
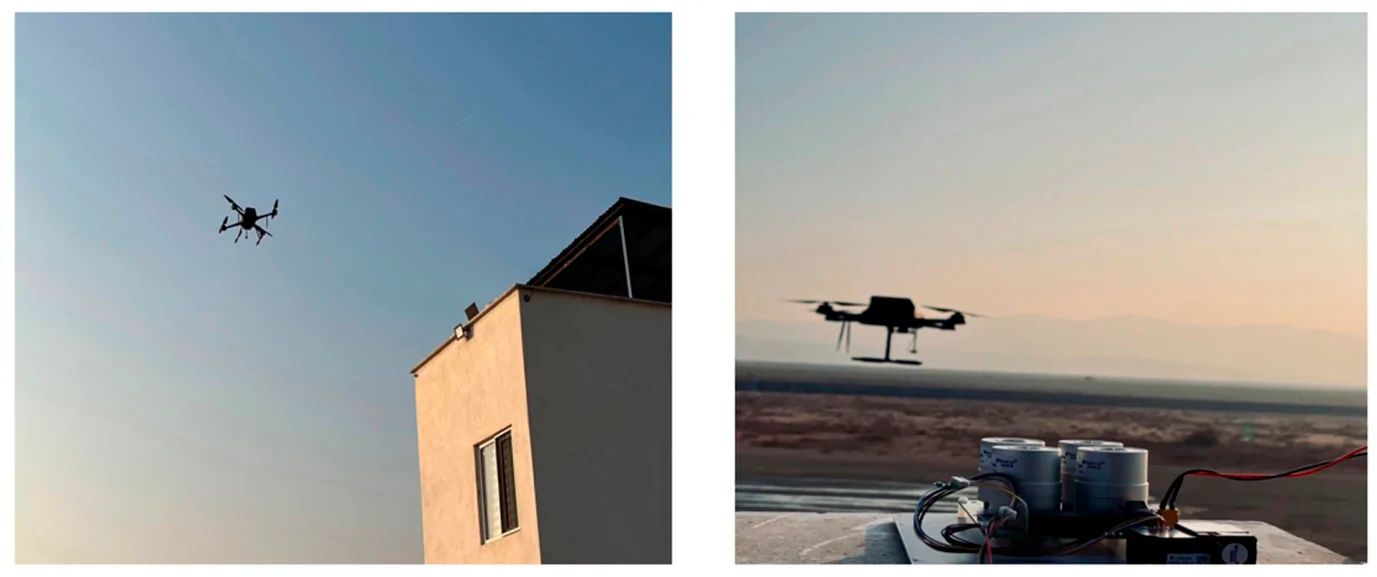
Photograph of the hover co-location test setup, showing the fixed reference and UAV-mounted sensors during the Zone 4 hover test.

The reference platform used throughout this study, for both the analytical examples and the experimental validation below, is a 21-inch-propeller quadrotor (Section 5.2.1; Table 4). Two identical sensor units were used for the flight tests: an all-in-one module (ZEHS04, Zhengzhou Winsen Electronic Technology Co., Ltd., Zhengzhou, China) reporting CO, NO_2_, O_3_, PM_2.5_, and PM_10_ in µg/m^3^ over an active-upload RS485 interface [39,40] (Figure 6), with one unit at the Zone 4 position (Section 6.2) and the second held stationary as a fixed ground reference (Figure 7). The O_3_ channel of the ZEHS04 unit used in the flight tests is electrochemical rather than UV-photometric; accordingly, the O_3_ flight-test results are interpreted here as evidence regarding placement-related measurement behavior, not as validation of the UV-photometric O_3_ response assumptions used in the framework.

### 6.2. Sensor Placement Design

Building on the contamination zones (Section 5.5) and measurement bias (Section 5.6), this section gives engineering guidelines for sensor placement to minimize exposure to turbulent rotor wake. Minimum height and lateral offset values derive from momentum-theory models calibrated against published data [9,18,19].

The framework identifies sensor placement above the rotor plane, within Zone 4 (Table 5), as the analytically optimal configuration for minimizing rotor-induced measurement bias. The framework’s minimum height criterion derives from the empirical TI recovery distance in Section 5.3, adopting the lower (minimum-sufficient, not the most conservative) bound of the 3–5 rotor-diameter range for a compact design footprint; platforms prioritizing a wider safety margin over compactness should instead adopt the upper bound (5D):

(12) $$H_{\mathrm{mast}}>3\cdot D$$

Stated more systematically, the placement problem is one of choosing mast height H_mast_ and lateral offset Δx, for a given propeller diameter D and operating flight speed V, so as to minimize the resulting measurement bias: qualitatively, Bias = f(H_mast_, Δx; D, V, TI), where D and Equation (1) set the downwash field that TI (Section 5.3) is derived from, H_mast_ and Δx position the sensor within that field via the zone boundaries of Table 5, and V enters through the speed-dependent terms of Equations (8) and (9). Equation (12) (H_mast_ > 3D) and the Δx ≥ 0.10–0.15 m criterion above are therefore best read as one feasible region of this broader design space—the minimum-sufficient point for a compact, low-speed configuration—rather than a single universal optimum. Table 8 tabulates two further points in this space (quadrotor and hex-rotor configurations) rather than a continuous solution; formally solving for the bias-minimizing (H_mast_, Δx) pair as a function of D and V across the full operating envelope would require a numerical optimization exercise that is beyond the scope of the present analytical framework and is not attempted here.

In this study, the gas sensor unit described in Section 6.1 was mounted directly above the rotor disks, within the Zone 4 region, integrated into the airframe rather than raised on a separate elevated mast. Horizontal offset from the nearest blade tip satisfied Δx ≥ 0.10–0.15 m to avoid blade tip vortex contamination. Tier II (electrochemical) bias estimates carry a roughly four-fold uncertainty from the unresolved k_flow_ coefficient (Table 6 notes), so mast-height guidance for these sensors should size conservatively toward the k_flow_ upper bound pending wind-tunnel calibration. Sensors between adjacent rotor disks should use the Zone 2b inter-rotor criteria (Table 5) rather than Zone 4 until clearing the merged-wake footprint at ~2 rotor diameters. Table 8 gives the framework’s recommended mounting parameters for two representative configurations that use an elevated-mast design to satisfy the Zone 4 height criterion; this study’s direct above-rotor integration achieves the same criterion without a separate mast. These mast-height figures derive from the aerodynamic clearance criterion alone. A rigid boom of this length also shifts the centre of gravity and increases moment of inertia on a sub-2 kg airframe—a flight-stability trade-off not analysed here that needs separate verification for any physical mast design. (Table 8 and Section 6.2 describe the framework’s general design-space recommendation, independent of the specific hardware used for the Section 7 flight tests).

The mounting parameters in Table 8 differ substantially between the 21-inch quadrotor and 15-inch hex rotor, reflecting the strong dependence of downwash velocity and TI on propeller diameter (Table 4). The additional PM sensor clearance (≥0.45 m vs. ≥0.35 m) is consistent with Tier I sensitivity (Table 6), giving platform designers a more structured, physics-grounded starting point than largely empirical prior practice.

Table 9 compares placement scenarios, estimated bias, and recommended actions.

The predicted consequences of suboptimal placement are most severe for below-disk and at-disk-plane configurations (Zones 1–2), with estimated PM bias of 30–60%. The priority classifications in Table 9 give platform designers a structured triage: critical-priority cases (Zone 1) require hardware redesign, high-priority cases (Zone 2) also warrant geometric redesign, and medium-priority cases (Zone 3) may be addressed through bias correction alone.

Table 10 consolidates six non-optimal design and operational parameters against their optimization approach and estimated bias before and after.

### 6.3. Experimental Setup: Hover and Constant-Speed Flight Tests

#### 6.3.1. Hover Co-Location Flight Test Procedure

A hover co-location flight test provided direct experimental support for the framework’s central placement prediction (Section 5.5.2, Zone 4), using the paper’s 21-inch-propeller reference quadrotor (Section 5.2.1, Table 4). Two identical sensor units were used: one fixed at a reference height of 15 m above ground level and one mounted on the UAV at the same height, co-located and cross-calibrated beforehand so any pre-existing offset would not be conflated with rotor-induced bias. The UAV was flown to a stable hover with the sensor positioned above the rotor plane, within Zone 4, at the same 15 m altitude as the fixed sensor and at a lateral offset from it—following the qualitative above-rotor, Zone 4 criterion rather than the specific elevated-mast height calculated in Section 6.2. The fixed sensor kept sampling from its stationary position throughout. Both streams were logged with independent timestamps. The ZEHS04 sensor operated in its default active-upload mode, providing concentration measurements at a 1 s output interval [40]. For temporal synchronization, the fixed-reference and UAV-mounted datasets were independently aggregated to a common 2 s time base using arithmetic averaging within each 2 s bin, following standard time-series alignment practice [41]; only timestamps common to both aggregated datasets were retained for comparison.

#### 6.3.2. Constant-Speed Flight Test Procedure

To assess the framework’s prediction that motion-induced bias increases with forward flight speed (Section 5.7, Equation (9)), a second flight test used the same platform, sensor pair [40], and above-rotor Zone 4 mounting position described in Section 6.3.1, with the UAV flown at a constant forward speed of 5 m/s instead of hover. As before, the fixed reference remained stationary while the UAV traversed Zone 4, the pre-test cross-calibration offset was applied identically, and both streams were logged with independent timestamps and independently aggregated to a common 2 s time base using arithmetic averaging within each 2 s bin, as in Section 6.3.1; only timestamps common to both aggregated datasets were retained for comparison.

## 7. Results

### 7.1. Experimental Validation: Hover Co-Location Flight Test

**Figure 8:**
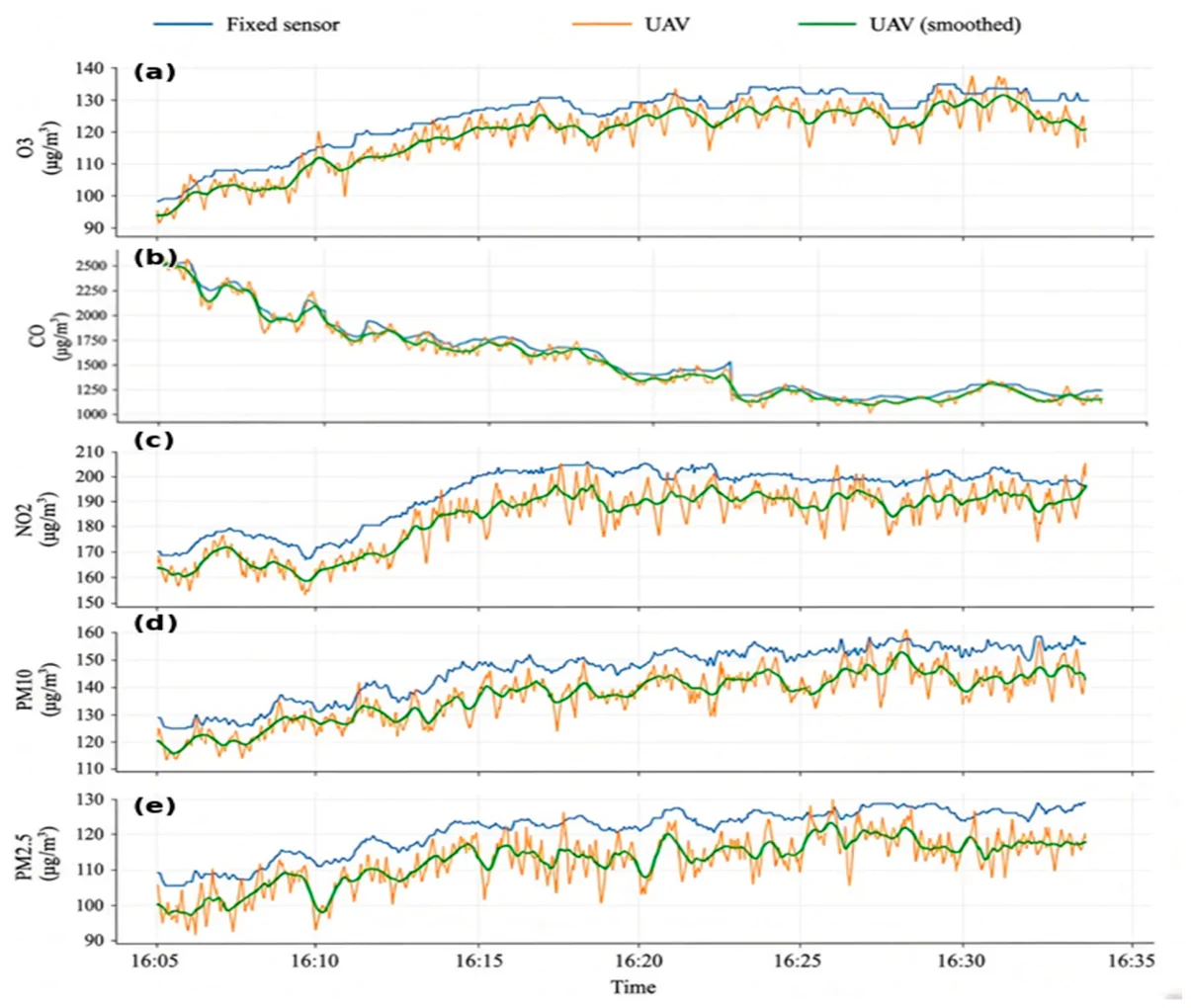
Comparison of pollutant concentrations from the fixed reference and UAV-mounted sensor during the Zone 4 hover test: O_3_ (**a**), CO (**b**), NO_2_ (**c**), PM_10_ (**d**), PM_2.5_ (**e**). The raw UAV signal (orange) and its smoothed trace (green) are shown against the fixed reference (blue).

Across the five pollutants compared, the UAV-mounted sensor tracked the fixed reference closely, with a systematic bias of 3% (CO) to 10% (PM_2.5_) and correlation coefficients of 0.82–0.99. The corresponding time series are shown in Figure 8. This mean-ratio bias is time-averaged and signed, so it can mask compensating over- and under-estimation; the MAE and RMSE in Table 11 give a more complete picture than the percentage alone. A further caveat applies to the correlation coefficients: the electrochemical sensors’ T90 response times (tens of seconds) are slower than the analysis time base, so consecutive samples are likely autocorrelated. High r values should be read as shared low-frequency trend-tracking rather than fine-grained tracking fidelity, making MAE/RMSE a more response-time-independent basis for agreement. This is consistent with the framework’s Zone 4 prediction (Section 5.5.2, Table 5) that the region above the rotor plane gives near-ambient conditions and matches the order of magnitude of the near-zero hover-mode bias estimated in Table 9. The residual bias—higher than the idealized estimate but far below the 30–60% predicted for Zones 1–2—plausibly reflects sensor-to-sensor calibration offset, environmental drift, and residual low-level turbulence. Ambient wind speed and direction were not measured during this test. The framework’s own model predicts that ambient crosswind can add an estimated 5–15% turbulence-intensity increment at the sensor (Section 5.2.3)—a range that spans the entire observed hover bias—so an unmeasured wind contribution cannot be ruled out alongside the calibration-offset and residual-turbulence mechanisms above. This test establishes the hover-condition stage of the validation pathway (Section 5.1); Section 7.2 extends validation to forward flight, with wind-tunnel characterization across more propeller sizes a natural next step to broaden the framework’s validated range.

### 7.2. Experimental Validation: Constant-Speed Flight Test

**Figure 9:**
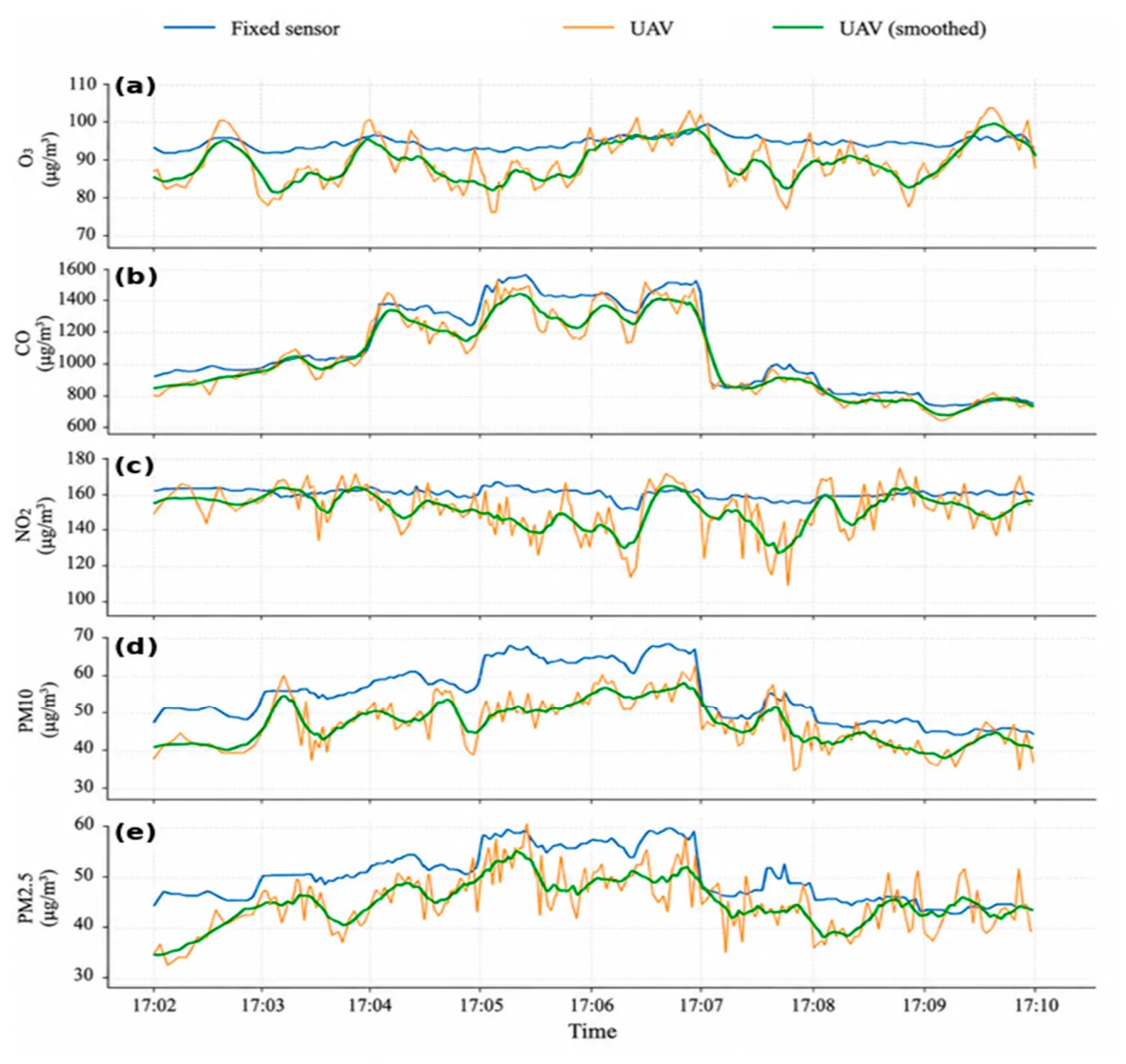
Comparison of pollutant concentrations from the fixed reference and UAV-mounted sensor during the Zone 4 constant-speed (5 m/s) flight test: O_3_ (**a**), CO (**b**), NO_2_ (**c**), PM_10_ (**d**), PM_2.5_ (**e**). The raw UAV signal (orange) and its smoothed trace (green) are shown against the fixed reference (blue).

Across the five pollutants, the UAV-mounted sensor showed a systematic negative bias during constant-speed flight, from 6.3% (NO_2_) to 14.9% (PM_10_). The corresponding constant-speed time series are shown in Figure 9, and agreement metrics are summarized in Table 12. This exceeds the 3–10% hover-mode bias (Section 7.1) for PM_10_ and PM_2.5_, consistent with the framework’s prediction that forward flight adds a motion-induced bias component (f_motion_(v), Equation (9)) atop the near-ambient Zone 4 baseline. Absolute errors tell a more nuanced story: PM_2.5_’s percentage bias rose (9.7% → 12.8%) while its absolute MAE actually fell (11.75 → 6.93 µg/m^3^)—a denominator artifact rather than necessarily a larger aerodynamic effect; PM_10_ shows the same pattern (MAE 11.00 → 8.34 despite higher % bias). Thus, the forward-flight evidence for a PM-specific motion component is directionally consistent but not unambiguous once baseline normalization is considered. NO_2_’s increase (6.2% → 6.3%) is marginal and within likely measurement noise, so this single test does not clearly confirm a motion-induced component for that pollutant. Correlation coefficients (r = 0.44–0.94) were markedly lower than in hover (0.82–0.99), especially for NO_2_ and O_3_, indicating greater variability from higher-frequency turbulence during translation. CO showed the smallest degradation (r = 0.94), possibly reflecting its slower electrochemical response smoothing high-frequency fluctuations, or CO’s more spatially uniform ambient background relative to NO_2_ and O_3_—the present tests cannot distinguish between these. As in the hover test (Section 7.1), ambient wind speed and direction were not measured during this flight; since forward motion changes the platform’s airspeed relative to any ambient wind, an unmeasured crosswind could also alter the effective advance ratio and wake tilt (Section 5.2.3) during translation, so the observed bias increase cannot be attributed to platform motion alone with full confidence. A related limitation: since the UAV moves at 5 m/s while the reference sensor stays fixed, the two were not always sampling the same air parcel, so some of the observed bias increase could reflect spatial heterogeneity along the flight path rather than motion effects alone; a co-located or tethered-reference design would be needed to isolate f_motion_(v) from this confound. These results extend the validation pathway (Section 5.1) to a single forward-flight speed; multi-speed campaigns remain necessary to parameterize Equation (9) for this platform. A further confound: reference PM_2.5_ fell from 121.2 to 49.3 µg/m^3^ (59%) between the hover and constant-speed sessions (CO and NO_2_ also lower, by 31% and 14%), so the two tests sampled different ambient regimes, not just different motion states. Since coincidence probability and sensor noise floor are themselves concentration-dependent, part of the bias increase attributed to motion may instead reflect this regime shift; matched-concentration repeat testing would be needed to separate the two effects.

### 7.3. Agreement Between Predicted and Observed Bias

The hover and constant-speed tests together support the framework’s central predictions. Observed hover-mode bias (3–10%, Section 7.1) falls within the near-ambient range predicted for Zone 4 and within the Section 5.8 uncertainty envelope. The bias increase under constant-speed flight (6.3–14.9%, Section 7.2) matches the direction and magnitude of the motion-induced term f_motion_(v) (Section 5.7, Equation (9)), though lower forward-flight correlation (r = 0.44–0.94 vs. 0.82–0.99 in hover) shows more variability than mean bias alone captures. These two tests offer encouraging, if still limited, empirical support.

## 8. Discussion

### 8.1. Implications for Monitoring Campaign Design

The results carry direct implications for UAV-based air quality monitoring campaign design. Hover mode provides conditions most conducive to accurate measurement: near-zero freestream velocity eliminates motion-induced bias (Table 7), and elevated mast mounting places sensors within Zone 4 (Table 5), outside the predicted influence of rotor wakes. Hover-and-sample strategies are recommended for applications requiring precise local concentration data, with forward-flight transit sampling reserved for spatial reconnaissance.

The propeller size comparison in Table 4 shows Equation (12)’s mast height requirement (H_mast_ > 3D) applies at every size, scaling from ~0.76 m for 10-inch propellers to 1.90 m for 25-inch propellers. Mast heights near or exceeding ~1 m (13-inch propellers and larger) may exceed packaging constraints on compact platforms, increasing reliance on bias correction (Section 5.9). Platforms with 10-inch propellers at moderate RPM are predicted to attain PM bias of 10–18% with elevated placement, versus up to 25% for 13-inch platforms.

The turbulence source classification in Table 3 identifies hybrid turbulence during forward flight as the worst case, with estimated PM bias of 35–55%. Campaigns should therefore prioritize hovering protocols for Tier I and II pollutants wherever feasible, using forward-flight data only for spatial pattern mapping at reduced accuracy expectations.

The optimization scenarios in Table 10 show each of the six design parameters contributes independently to bias, with combined optimization reducing bias from 30–60% (worst case) to ±5–15% (optimized, with correction). A one-at-a-time comparison ranks mounting-height placement as the dominant lever—Zone 1 to Zone 4 changes PM bias by ~20–50 points (Table 10)—well ahead of the motion term (≤12 points, Table 10) and environmental term (≤15 points, Section 5.8). Hardware redesign should therefore be prioritized ahead of bias correction alone, though a full variance-based sensitivity analysis (e.g., [42]) remains a future priority.

### 8.2. Comparison with Previous Approaches

Table 13 positions the present framework relative to key prior studies. Prior work has addressed individual components of the problem in isolation: [20] developed a specialized sampling system with limited generalizability across pollutant classes, and aerodynamic CFD studies [10,11] provided high-fidelity wake characterization without connecting aerodynamic outputs to sensor bias estimation or correction strategies. The present framework’s contribution is the integration of these previously disconnected components into a single, analytically grounded methodology.

Unlike the experimental studies in Table 13, the present framework provides analytical estimates from literature-constrained models rather than direct measurements—an intentional trade-off favoring broad, physics-grounded applicability over site-specific accuracy, with limitations discussed below.

### 8.3. Limitations and Future Research Directions

The boundaries of the current framework define a clear, tractable research agenda: each constraint identified below corresponds to a specific future-work direction that, if addressed, will progressively strengthen the framework’s predictive capability and operational applicability.

The most significant boundary of the present work is the absence of a full original experimental UAV campaign: most quantitative estimates derive from analytical models calibrated against published literature, consistent with the framework’s role as a design and planning tool. The highest-priority future direction is therefore a systematic experimental validation programmed following the roadmap in Section 5.1, yielding platform-specific coefficients for quantitative application of the bias correction formulation (Equations (10) and (11)) with traceable uncertainty bounds.

The aerodynamic analysis relies on actuator disk momentum-theory approximations, validated qualitatively against referenced studies. These are efficient, first-order estimates suitable for design guidance, but they do not capture unsteady blade-vortex interaction, rotor-fuselage interference, or blade-wake impingement—platform-specific RANS or DES/LES simulations could resolve these to refine the Section 5.5 zone boundaries. The classification and mast-height guidelines also derive from free-air momentum theory and do not hold within ground effect (altitude below ~2 rotor diameters), where downwash recirculation can transport surface-level pollutants toward the sensor and reduce induced velocity by an estimated 20–40%. Practitioners should avoid sampling below ~1–2 m AGL during takeoff and landing, pending a dedicated ground-effect correction model.

The empirical model coefficients require platform- and sensor-specific experimental identification before quantitative operational use. The propagated uncertainty (±10–15%, Section 5.8) is appropriate for a design-planning tool and can be reduced through targeted wind tunnel calibration of each sensor model—a tractable laboratory step ahead of prototype field deployment.

The framework assumes a spatially homogeneous ambient pollutant field (Section 5.1, Assumption 3); atmospheric stability, terrain-induced turbulence, and temperature inversions may substantially alter real-world accuracy beyond what these captures. A valuable future direction is incorporating stability-dependent turbulence parameterizations and terrain roughness corrections, following urban boundary layer studies such as Ahlawat et al. [7].

The two-stage correction model (Equation (10)) assumes linear superposition of motion-induced and environmental bias factors; non-linear interactions, particularly at high speed in gusty conditions, are not captured. Machine-learning models—random forest or neural network, as for low-cost sensors by Zimmerman et al. [43]—could be trained on simultaneous UAV and reference measurements to capture such non-linear dependencies, potentially reducing residual uncertainty below the ±10–15% bound.

These limitations point to a consistent set of priorities: wind tunnel and multi-speed flight validation to establish platform-specific coefficients, higher-fidelity CFD simulation to refine the aerodynamic parameterization, and machine-learning correction models to capture non-linear effects beyond the current analytical form. A further priority is characterizing vertical-motion effects during hover (Section 5.5.4), since the mast-height criterion (Equation (12)) assumes a fixed sensor altitude relative to the rotor plane; residual vertical velocity during hover could transiently alter this relative position and is not characterized by the present flight tests. Each builds directly on the hover and constant-speed co-location tests reported in Section 7.1 and Section 7.2, together defining the path toward an experimentally validated, operationally deployable system.

### 8.4. Practical Applicability and Deployment Recommendations

Both UAV platform designers and monitoring campaign operators are intended beneficiaries. Deployment guidelines for four representative scenarios follow; all values are analytical estimates requiring platform-specific calibration before use.

Small UAVs (≤4 rotors, propeller diameter ≤ 25 cm): an elevated mast at ≥0.75 m above the highest rotor plane (≥3 rotor diameters, Equation (12)) is recommended, with the f_motion_ correction (Equation (9)) applied at flight speeds above 5 m/s.

Large UAVs (≥6 rotors, propeller diameter ≥ 33 cm): sensor placement at or below the rotor plane is contraindicated; lateral boom placement beyond the rotor disk radius is recommended, with multi-speed calibration flights essential for accurate correction model parameterization.

PM sensor deployments (PM_2.5_, PM_10_): optical particle counters are expected to be particularly sensitive to inlet flow velocity fluctuations, so hovering at fixed waypoints is preferable to continuous transects for absolute concentration measurement, eliminating the dominant motion-induced bias (Table 7).

Gas sensor deployments (NO_2_, CO, O_3_, VOCs): the two-stage correction combining f_motion with f_env (Equation (10)) is recommended for all electrochemical gas sensor data, anchored by ground-based co-location against a reference instrument during takeoff and landing. Coefficients in Table 6 and Table 9 are illustrative; values for each sensor model must be established experimentally per Table 10.

Vertical profiling deployments: when sampling at multiple altitudes to characterize vertical pollutant gradients, a step-and-hold protocol—pausing at each target altitude before recording, rather than sampling during continuous ascent or descent—follows the same rationale as the fixed-waypoint recommendation above for PM sensors, and additionally addresses the vertical-motion uncertainty discussed in Section 5.5.4. Given the electrochemical sensors’ T90 response times of tens of seconds (Section 7.3), a dwell time of comparable order at each altitude would allow the reading to settle toward ambient conditions at that height rather than reflect a transitional mixture; the specific altitude increment and dwell duration would need to be established for the sensor and platform combination in use.

## 9. Conclusions

This paper develops a physics-informed methodology for estimating how rotor-induced turbulence affects air quality measurement accuracy in multi-rotor UAVs. Its main contribution is integrating previously disconnected components—rotor aerodynamics, turbulence generation, sensor disturbance, pollutant-specific bias, and correction strategy—into a single, physics-constrained foundation for UAV sensor integration design, uncertainty estimation, and campaign planning. Hover and constant-speed co-location tests on the reference 21-inch quadrotor (Section 7.1 and Section 7.2) give initial experimental support for the central Zone 4 placement prediction and its motion-dependent bias trend; further work should extend this to other platforms, flight speeds, and pollutant classes. The main conclusions:

- Three turbulence sources (propeller-induced, wind-induced, and hybrid combined) were characterized, and four sensor contamination zones defined from momentum-theory wake analysis (Table 3 and Table 5). The region above the rotor plane (Zone 4) is consistently predicted as the most favorable measurement location, qualitatively supported by several independent experimental studies [18,19,20].
- A pollutant-dependent sensitivity classification and a speed- and environment-dependent correction methodology (Equations (9)–(12)) identify propeller size, flight speed, and sensor mounting geometry as the dominant design parameters governing measurement bias (Table 3 and Table 8 and Table 9).
- A physics-informed bias correction formulation (Section 5.9, Equations (10) and (11)) was proposed as an engineering pathway for reducing residual measurement uncertainty, with platform-specific identification of its coefficients as the necessary next step beyond the validation reported in Section 7.1 and Section 7.2.
- Hover and constant-speed co-location flight tests (Section 7.1 and Section 7.2, Table 11 and Table 12) compared a UAV-mounted sensor against a synchronized fixed reference for NO_2_, CO, O_3_, PM_10_, and PM_2.5_ under Zone 4 conditions. Bias was 3–10% in hover (r = 0.82–0.99), rising to 6–15% at 5 m/s forward flight (r = 0.44–0.94)—directionally consistent support for both the central placement prediction and the motion-dependent bias trend. But the lower end of the forward-flight correlation range falls below typical instrument-validation thresholds, and with only a single platform, two flight conditions, and five of nine target pollutants tested, this should be read as preliminary rather than confirmatory evidence.
- A propagated uncertainty analysis (Section 5.8) bounded the framework’s overall analytical uncertainty at approximately ±10–15% under the stated modelling assumptions, a range considered appropriate for design and planning purposes but insufficient for regulatory-grade or high-precision operational monitoring without platform-specific calibration.

This methodology is meant to support UAV platform design, sensor placement decisions, and calibration campaign planning before field deployment—a foundation for the experimental validation that should follow, not a substitute for it. Its main practical value is helping practitioners prioritize the most influential design parameters—mounting height, propeller size, flight speed, and inlet conditioning—before committing resources to field campaigns. Future research should prioritize wind-tunnel characterization, co-location experiments, and platform-specific CFD simulation (Section 8.3) to establish the coefficients needed for operational use. The framework is best read as a pre-deployment design and calibration planning tool, not a substitute for experimental qualification of UAV-mounted sensing systems.

## Figures and Tables

**Table 1:** Comparative summary of key prior studies in UAV-based air quality monitoring. Reference numbers correspond to the full citations, including DOIs, listed in the References section.

Reference	UAV Type	Sensor Type	Pollutants	Main Finding/Contribution	Key Limitation
Toosi et al. [5]	VTOL + Fixed-wing (review)	Multiple	NO_2_, PM_2.5_, PM_10_, CO, O_3_	UAVs superior for local-scale monitoring; AI/IoT integration transforms data processing	Review only; no quantification of rotor-induced measurement bias
Ahlawat et al. [7]	Multirotor VTOL	OPC (PM-LCS)	PM_2.5_, PM_1_	Urban haze 60% higher at 100 m vs. ground; model underestimates by 52.6%	No analytical correction framework for rotor-induced sensor error
Narayan [16]	Multiple (review)	Multiple	Multiple	Downwash, isokinetic, and mount location identified as primary accuracy determinants	Qualitative only; no unified quantitative error model
Kartal et al. [10]	Quadcopter	Thrust + CFD	N/A (aerodynamics)	Experimental + CFD aerodynamic characterization at varying RPM	No link to sensor measurement bias or pollutant-specific effects
Liu et al. [11]	Multirotor formation	Wind tunnel + CFD	N/A (flow)	Rotor separation strongly affects thrust and wake interaction structure	Wake characterized without pollutant transport or sensor error analysis
Shouji et al. [9]	Agricultural drone	Hot-wire CTA	N/A (downwash)	Spiral rotational downwash; turbulence characterized at 30 kHz sampling	Single rotor only; no multi-rotor interference or sensor placement analysis
Villa et al. [6]	VTOL + Fixed-wing (review)	Multiple	Multiple	First systematic UAV air quality review; 60 studies analyses	Predates detailed rotor aerodynamic error quantification
Wang et al. [18]	Quadcopter	OPC	PM_2.5_, PM_10_	Propeller disturbance correction; tower-reference comparison	Empirical correction without generalized analytical framework
Brosy et al. [20]	Hexacopter	NDIR tube sampler	CH_4_	CH_4_ gradients resolved; optimized inlet placement demonstrated	Validated for CH_4_ under stable conditions; multi pollutant use remains unverified, though flow conditioning may extend to other gas sensors.
Pochwala et al. [19]	Multirotor	OPC, EC	PM_2.5_, O_3_	Above-rotor placement associated with reduced error; field tests in Poland	No analytical model linking turbulence intensity to error magnitude

**Table 2:** Different types of UAV platforms and their specifications [5].

No.	Platform Type	Presentation	Pros	Cons
1	Fixed-wing	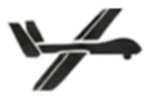	High endurance; high flight altitude; high payload capacity	Requires launcher or human effort for launching; needs runway for landing; poor maneuverability
2	Fixed-wing	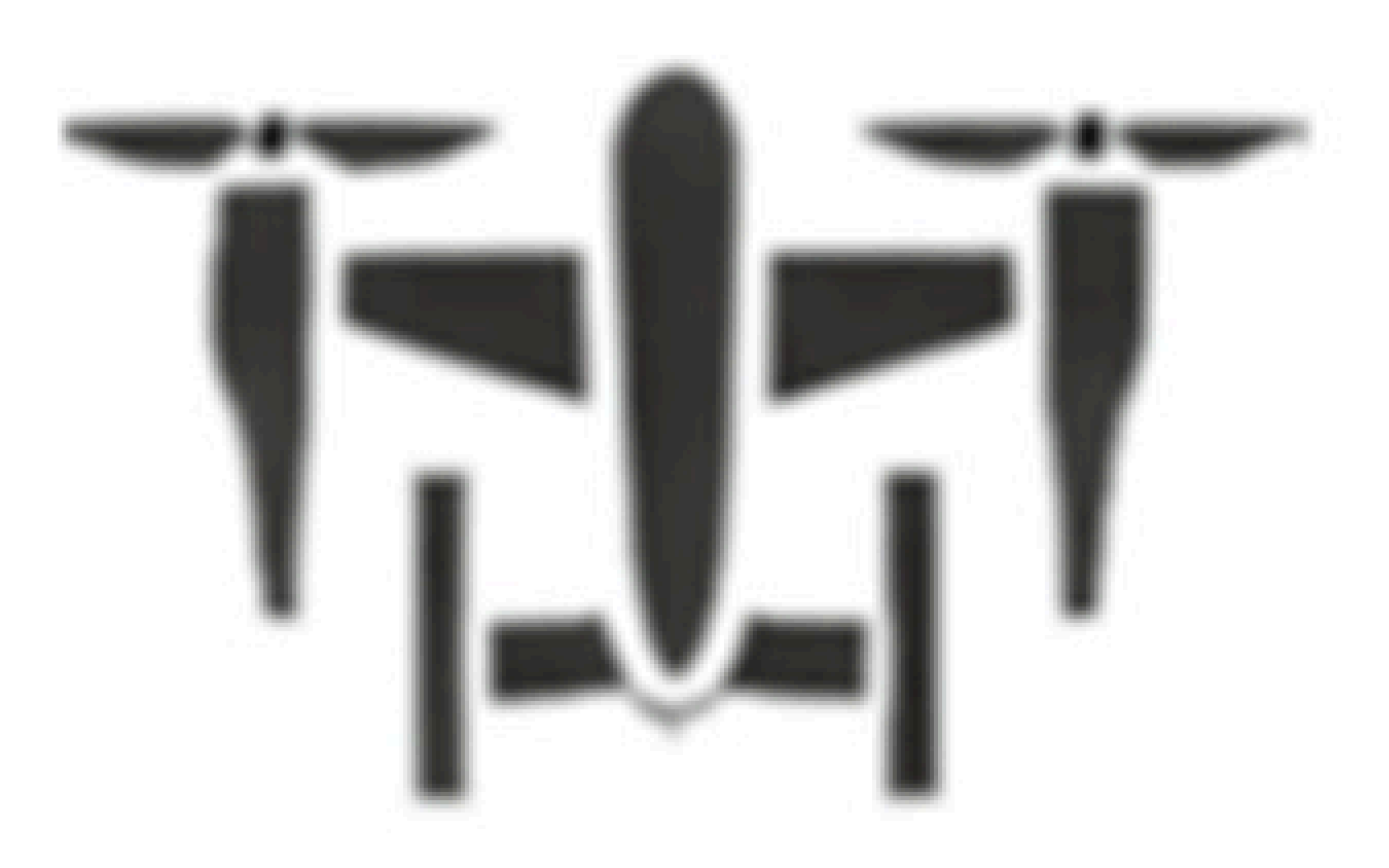	High endurance and payload capacity; VTOL and low altitude hovering; combines fixed wing efficiency with rotary wing flexibility.	Expensive; complex technology
3	Rotary-wing multi rotors	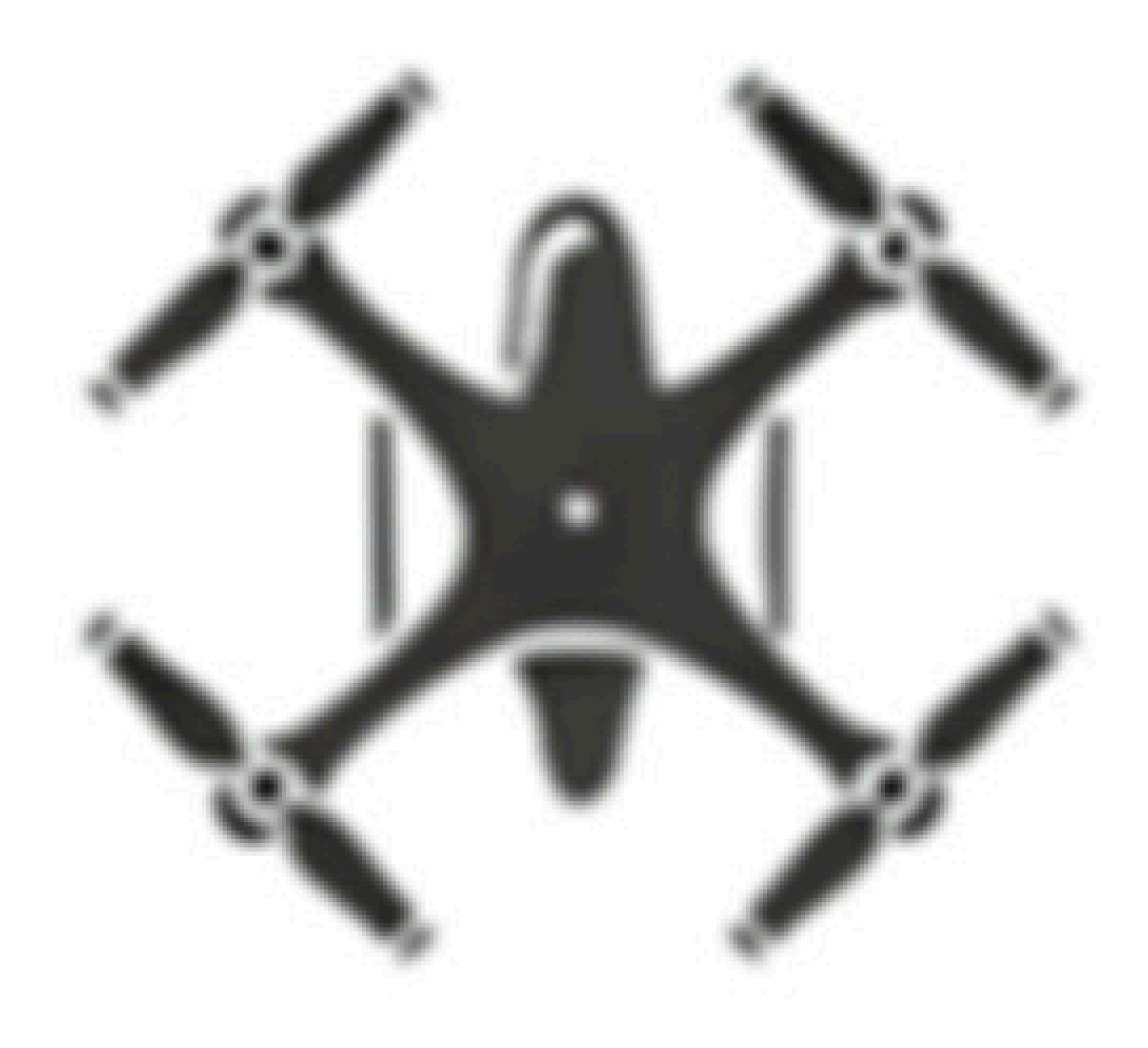	Low flight altitude; VTOL capability; hovering capability; lightweight; good maneuverability; inexpensive	Low endurance; limited payload capacity; susceptible to wind; strong downwash effect
4	Rotary-wing unmanned helicopter	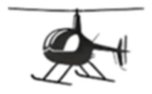	Low flight altitude; VTOL capability; good maneuverability; hovering capability; lightweight	Low endurance; limited payload capacity; susceptible to wind
5	Unmanned airship and balloon	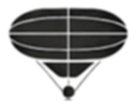	Hovering capability; high payload capacity; lightweight; VTOL capability	Poor maneuverability

**Table 3:** Classification of turbulence sources in multi-rotor UAVs, predicted airflow effects, and estimated impact on pollutant measurements. Values are analytically derived and require experimental verification.

Turbulence Source	Generation Mechanism	Predicted Airflow Effect	Pollutant Transport Impact	Est. Measurement Error	Design Implication
Propeller-Induced	Rotating blades accelerate air downward, creating pressure differential below rotor disk	Downwash jet 8–14 m/s; blade passage pressure spikes at BPF	Predicted dilution of PM; reduced analyte contact time for electrochemical gas sensors	PM: 30–60%; Gas: 10–35%	Elevate sensors above rotor plane (Zone 4); apply f_motion_ correction
Rotor Wake Turbulence	Blade tip vortices shed at ~0.8–1.2 rotor radii from disk center	TI = 25–40% in tip vortex annulus; unsteady velocity fluctuations	Particle trajectory deflection; OPC coincidence errors; elevated gas noise	PM: 15–30%; Gas: 5–15%	Avoid Zone 2 (blade tip annulus); maintain ≥0.15 m lateral offset from tip
Rotor-to-Rotor Interaction	Adjacent rotor wakes merge; aerodynamic interference amplifies turbulence	Complex velocity field; amplified TI = 20–35% in overlap region	Non-uniform plume transport; concentration gradients across platform	PM: 10–25%; Gas: 8–20%	Maintain ≥2 rotor-diameter spacing; account for geometric bias in correction
Wind-Induced (Ambient)	Environmental crosswind interacts with UAV body and rotor wakes	Asymmetric distortion of downwash; increased effective TI on leeward side	Windward/leeward sensor asymmetry; variable and attitude-dependent sampling rate	PM: 5–20%; Gas: 5–15%	Monitor wind speed/direction in flight metadata; apply k_W_ correction (Equation (11))
Hybrid (Wind + Propeller)	Combined effect of ambient wind and propeller thrust during forward flight	Tilted wake governed by advance ratio J = V/nD; worst-case disturbance field	Maximum predicted dilution; concurrent motion and wind errors at 20–40 km/h	PM: 35–55%; Gas: 15–35%	Prefer hover-and-sample for PM_2.5_; limit transit speed ≤10 km/h for Tier I pollutants

**Table 4:** Effect of propeller diameter on predicted downwash velocity, airflow volume, and turbulence intensity, with associated bias and disturbance level for representative propeller sizes.

Propeller Size	Typical RPM	Predicted Downwash (m/s)	Est. Airflow (m^3^/min)	Est. TI—Central Column (%)	Predicted Effect on Measurement	Disturbance Level
10 in (254 mm)	5000–9000	3.2–4.6	0.6–1.1	10–18	Low dilution; PM error <15%; gas error <8%.	Low
13 in (330 mm)	3500–6500	4.3–6.0	1.0–1.8	12–22	Moderate PM dilution; PM error 15–25%; gas error 8–15%.	Low–Medium
15 in (381 mm)	2800–5000	5.3–7.1	1.6–2.7	15–28	PM error 20–35%; gas error 10–20%; hover sampling recommended	Medium
17 in (432 mm)	2200–4000	6.0–8.2	2.0–3.5	18–30	Predicted PM error 25–40%; elevated mount analytically recommended	Medium–High
21 in (533 mm)	1500–2800	6.7–8.9	2.8–5.3	20–35	PM error 30–45%; mast ≥1.6 m required (Equation (12)).	High
25 in (635 mm)	1000–2000	7.8–10.3	3.9–6.7	22–40	PM error 35–55%; boom mount critical; full correction required.	Very High

**Table 5:** Contamination zone classification for multi-rotor UAVs: framework-predicted characteristics and sensor placement implications. Zone boundaries and TI values are constrained by experimental data from [9,18,19].

Zone	Location	Mean Velocity	TI (%)	Dominant Predicted Effect	Mounting Suitability
Zone 1 (Primary Downwash)	Below rotor disk, 0–3 rotor diameters downstream	8–14	15–25	Strong particle and gas transport; maximum dilution.	Analytically unsuitable for all pollutant types; avoid entirely
Zone 2 (Blade Tip Vortex)	Annular region, 0.8–1.4 radii outboard, 0–2 diameters below disk	4–10	25–40	Centrifugal particle deflection and anisokinetic sampling bias; elevated noise floor	Not recommended; both aerosol and gas errors analytically significant
Zone 2b (Inter-Rotor Interference)	Between adjacent rotor disks, within ~2 rotor diameters above disk plane	4–10	20–35	Merged wake turbulence from adjacent-rotor interaction, structurally distinct from single-disk Zones 1–2 [11]	Not recommended; risk of misclassification as Zone 4 if inter-rotor footprint is not identified separately
Zone 3 (Lateral Induced Flow)	Outboard and above rotor plane; 2–5 m/s lateral velocity component	2–5	8–15	Moderate predicted concentration error; variable with flight heading	Marginally acceptable with correction applied; extended mast preferred
Zone 4 (Clean Air Region)	Above rotor plane, >3× rotor diameter height (Equation (12))	<2	<5	Near-ambient conditions predicted; minimal aerodynamic disturbance from rotors	Predicted optimal location—recommended for scientific-grade measurements

**Table 8:** Recommended sensor mounting parameters for representative multi-rotor UAV configurations (hex rotor assumed at ~1.6 kg total mass, applying Equation (1) identically to the quadrotor case). Values are analytically derived and require platform-specific verification via hover co-location experiments.

Parameter	Quadrotor (21-Inch)	Hex Rotor (15-Inch)	Notes
Predicted downwash velocity (m/s)	~5.18	~6.2	From Equation (1); per-rotor thrust [24]
Est. TI—tip vortex (%)	25–40	25–40	Peak TI in Zone 2 annulus [9]
Est. TI—Zone 4 sensor position (%)	<5	<5	Analytically predicted above rotor plane
Minimum mast height (m)	1.55–1.65	1.10–1.20	Above highest rotor plane; from Equation (12)
Min. horizontal offset from blade tip (m)	0.10–0.15	0.07–0.11	Analytical estimate; avoid Zone 2
Recommended boom length—gas sensors (m)	1.60	1.15	Vertical extension above airframe
PM sensor mast—additional clearance (m)	≥1.65	≥1.20	PM Tier I most sensitive; extra clearance recommended
Sensor inlet orientation	0° (vertical, upward)	0° (vertical, upward)	Sample ambient air above rotor wake
ASC laminarization tube length (mm)	80–150	80–150	Settling chamber design guideline (Section 8)
ASC inner diameter (mm)	30–50	30–50	≥3× sensor inlet diameter

**Table 9:** Comparison of non-optimal and optimal sensor placement scenarios, with framework-derived bias estimates and recommended actions. Ranges are physics-informed model predictions, not measured outcomes.

Placement Scenario	Zone	Turbulence Level	Estimated Measurement Error	Recommended Action	Priority
Below rotor disk (body-integrated sensor)	Zone 1	TI 15–25%	PM: 30–60% est. underread; Gas: 20–40% est. underread	Hardware redesign is strongly recommended; correction alone may be insufficient	Critical
At rotor-plane level (body-mounted)	Zone 2	TI 25–40%	High variance; PM cycle error est. >15%; baseline drift in EC sensors	Not recommended—blade tip vortex is predicted to cause unstable readings	High
Aft of CG on multirotor body	Zone 3	TI 8–15%	PM: 10–25% est. underestimation; Gas: 5–15% est.	Marginal—apply correction factor; extended mast preferred	Medium
Elevated boom, ≥0.4 m above rotor plane	Zone 4	TI < 5%	PM: <10%; Gas: <8% after correction (analytical est.)	Predicted optimal—recommended for scientific campaigns	Low
Lateral boom, ≥0.15 m from blade tip	Zone 4 (lateral)	TI 5–12%	PM: <15%; Gas: <10% (analytical est.)	Acceptable—monitor attitude-dependent variability	Low–Medium

**Table 10:** Non-optimal versus optimized conditions for multi-rotor UAV air quality measurement, with analytically estimated error ranges. Values are model predictions from Section 5’s equations and literature constraints, not measured outcomes, and require platform-specific calibration before deployment.

Optimization Parameter	Non-Optimal Condition	Analytically Estimated Error Range (Non-Opt.)	Optimized Condition	Expected Residual Bias Range After Framework-Based Optimization
Sensor mounting height	At rotor plane or below (Zone 1–2)	PM: 30–60%; Gas: 20–40%	Elevated mast ≥3 rotor diameters above rotor plane (Zone 4)	PM: <10%; Gas: <8%
Propeller size	25-inch props; downwash 11–14 m/s	PM: 35–55%; Gas: 15–35%	Smaller rotors (10–13 in) or increased rotor count	PM: 15–25%; Gas: 8–15%
Flight speed	60 km/h forward flight	PM: 45%; Gas: 35%	Hover-and-sample or reduced speed (≤10 km/h)	PM: <8%; Gas: <6%
Inlet conditioning	No stabilization; direct ambient exposure	TI at sensor 25–40%; high variance	ASC with mesh + laminarization tube (L/D ≥ 2)	TI at sensor predicted <5%; more stable readings
Bias correction formulation	No correction applied	Raw error = full aerodynamic disturbance	Two-stage correction: f_motion_ × f_env_ applied in real time	Residual error est. ±5–15% PM; ±5–10% gas
Rotor-to-rotor spacing	Tight formation; strong inter-rotor interference	TI amplified 20–35%	Standard spacing ≥ 2 rotor diameters	TI reduced to 10–20%; more symmetric wake

**Table 11:** Agreement between the fixed reference and UAV-mounted sensor signal during the Zone 4 hover co-location test.

Pollutant	Mean—Fixed Sensor	Mean—UAV	Bias (%)	MAE	RMSE	Correlation (r)
NO_2_ (µg/m^3^)	196.4	184.2	6.2%	12.24	13.64	0.852
CO (µg/m^3^)	1573.2	1523.8	3.1%	55.30	65.99	0.993
PM_10_ (µg/m^3^)	139.5	128.6	7.9%	11.00	11.86	0.878
PM_2.5_ (µg/m^3^)	121.2	109.4	9.7%	11.75	12.68	0.815
O_3_ (µg/m^3^)	126.4	120.3	4.9%	6.45	7.38	0.933

**Table 12:** Agreement between the fixed reference and UAV-mounted sensor signal during the Zone 4 constant-speed (5 m/s) flight test.

Pollutant	Mean—Fixed Sensor	Mean—UAV	Bias (%)	MAE	RMSE	Correlation (r)
CO (µg/m^3^)	1088.4	984.7	9.5%	106.68	137.00	0.941
NO_2_ (µg/m^3^)	168.3	157.7	6.3%	13.52	17.47	0.435
O_3_ (µg/m^3^)	97.8	88.7	9.3%	10.27	12.00	0.449
PM_2.5_ (µg/m^3^)	49.3	43.0	12.8%	6.93	7.93	0.680
PM_10_ (µg/m^3^)	55.6	47.3	14.9%	8.34	9.42	0.765

**Table 13:** Positioning of the present framework relative to selected prior approaches in UAV air quality measurement research.

Study	Focus	Principal Limitation	Gap Addressed by This Work
Brosy et al. [20]	Specialized UAV gas sampling system design	Limited generalizability; no correction framework for multi-pollutant deployment	Generalized analytical design guidelines applicable across rotor configurations
Liu et al. [11]; Kartal et al. [10]	CFD and experimental wake characterization	No connection between aerodynamic outputs and sensor error or correction strategy	End-to-end analytical chain: rotor wake → turbulence → sensor bias → correction
This work	Unified physics-informed methodology: turbulence → bias → correction	Limited experimental campaign (2 flights, single platform); broader calibration needed	Integration of all prior components into a single analytically grounded design framework

## Data Availability

The data supporting the findings of this study are available from the corresponding author upon reasonable request.
